# Accurate detection of acute sleep deprivation using a metabolomic biomarker—A machine learning approach

**DOI:** 10.1126/sciadv.adj6834

**Published:** 2024-03-08

**Authors:** Katherine Jeppe, Suzanne Ftouni, Brunda Nijagal, Leilah K. Grant, Steven W. Lockley, Shantha M. W. Rajaratnam, Andrew J. K. Phillips, Malcolm J. McConville, Dedreia Tull, Clare Anderson

**Affiliations:** ^1^ School of Psychological Sciences and Turner Institute for Brain and Mental Health, Monash University, Melbourne, Australia.; ^2^ Cooperative Research Centre for Alertness, Safety and Productivity, Melbourne, Australia.; ^3^ Metabolomics Australia, Bio21 Molecular Science and Biotechnology Institute, Parkville, Australia.; ^4^ Division of Sleep and Circadian Disorders, Departments of Medicine and Neurology, Brigham and Women’s Hospital, Boston, MA, USA.; ^5^ Division of Sleep Medicine, Harvard Medical School, Boston, MA, USA.; ^6^ Centre for Human Brain Health, School of Psychology, University of Birmingham, Edgbaston, UK.

## Abstract

Sleep deprivation enhances risk for serious injury and fatality on the roads and in workplaces. To facilitate future management of these risks through advanced detection, we developed and validated a metabolomic biomarker of sleep deprivation in healthy, young participants, across three experiments. Bi-hourly plasma samples from 2 × 40-hour extended wake protocols (for train/test models) and 1 × 40-hour protocol with an 8-hour overnight sleep interval were analyzed by untargeted liquid chromatography–mass spectrometry. Using a knowledge-based machine learning approach, five consistently important variables were used to build predictive models. Sleep deprivation (24 to 38 hours awake) was predicted accurately in classification models [versus well-rested (0 to 16 hours)] (accuracy = 94.7%/AUC 99.2%, 79.3%/AUC 89.1%) and to a lesser extent in regression (*R*^2^ = 86.1 and 47.8%) models for within- and between-participant models, respectively. Metabolites were identified for replicability/future deployment. This approach for detecting acute sleep deprivation offers potential to reduce accidents through “fitness for duty” or “post-accident analysis” assessments.

## Teaser

A “blood-based” biomarker of sleep deprivation offers potential for future detection of sleep-related accidents.

## INTRODUCTION

Acute sleep deprivation is common in modern society, often due to competing demands for work, study, career, and recreational commitments. Globally, sleep deprivation is a substantial cause of motor vehicle crashes and occupational accidents and injuries (*1*, *2*), and the detrimental impact to human performance is widespread. For instance, sleep deprivation reduces the capacity to sustain attention and respond in a timely manner (*3*), ignore irrelevant stimuli to avoid distractors (*4*, *5*), and execute higher-order cognitions, including aspects of memory (*6*, *7*) and decision-making (*8*, *9*) and increases the likelihood of unintentional sleep episodes (e.g., microsleep) (*10*, *11*). Failure in any of these alertness and cognitive capabilities has severe consequences in safety-critical environments, such as transportation, health care, and high-consequence surveillance including air traffic control and automated operations. Despite these consequences, advances in managing the detrimental effects of sleep deprivation have been impeded by a lack of objective tools for detecting the sleep-deprived state (*12*).

Numerous tools have been developed to detect sleep loss and associated performance failure. These typically rely on well-established physiologic metrics such as pupillary stability, slow eye closures, or microsleep [e.g., (*10*, *13*, *14*)]. These metrics can be confounded by other factors that are associated with accidents, such as light (*15*) and adrenaline (*16*), and are rendered useless in the post-accident examination of an individual who has sustained a serious injury. Establishing a biological metric (“biomarker”) that accurately detects changing levels of sleep deprivation would offer considerable advantage in this respect.

A biological marker or biomarker of acute sleep deprivation needs to be responsive to extended wake (sensitive) while being unresponsive to other environmental factors (specific) and robust to interindividual variability (high predictive validity). Early research into the biological detection of sleep deprivation in humans identified salivary amylase as a potential candidate, where levels of mRNA representing amylase increased as a function of extended wake (*17*). Subsequent findings have been discrepant, however, potentially due to the confounds of stress and/or diurnal changes in amylase levels (*18*). More recently, transcriptomic studies demonstrate robust changes in gene expression as a function of sleep deprivation [e.g., (*19*–*22*)], providing several targets for sleep biomarker development. In a sophisticated multivariate search and validation approach, Laing *et al.* (*22*) demonstrated high accuracy (92%) for the classification of sleep deprivation when using a between-participant approach, and when detecting 7 to 8 hours awake versus 31 to 32 hours awake. Although still relatively high, accuracy reduced to 80% when comparing all hours above and below a 24-hour threshold, which may be more realistic in the real world. For their within-participant approach (a baseline adjusted sample), accuracy was slightly lower (74%) when comparing 10 to 11 hours awake versus 34 to 35 hours awake, and no comparative report was made in above/below 24-hour accuracy (*22*).

Changes in metabolite levels in serum and other biofluids provide a sensitive measure of the physiological state of an individual and their response to sleep deprivation (*23*–*26*), with several metabolites being identified as potential candidates for detecting sleep restriction and/or total deprivation (*27*, *28*). Recently, Depner *et al.* (*27*) demonstrated the capacity to detect chronic stable sleep restriction (5 hours of sleep for five consecutive days) with 74% accuracy using a combination of 65 metabolites, of which many were unidentified. While these data suggest that a suite of metabolite markers representing different biological mechanisms could achieve the accuracy and robustness required for a biomarker of sleep deprivation (*12*), several gaps remain, including the need for improved accuracy, and the identification and quantification of metabolites to aid replicability and translation to real-world environments.

In this study, we describe the development and validation of a metabolomic biomarker that accurately predicts how long an individual has been awake [time since wake (TSW)] and the presence of (>24-hour) sleep deprivation in young healthy adults with high accuracy. Polar plasma metabolites were detected using untargeted liquid chromatography–mass spectrometry (LC-MS) at different time points across 40 hours of continued wakefulness under controlled [constant routine (CR)] conditions. Machine learning identified a suite of metabolites that predicted sleep deprivation at the group and individual level and were validated in an unseen dataset. To promote the deployment potential for this biomarker, models were developed for a minimum number of (five) metabolites (to aid biosensor development), and biomarker candidates were identified (where possible) to enable future replication and validation under real-world conditions.

## RESULTS

Plasma samples were analyzed by Hydrophilic Interaction Liquid Chromotography (HILIC) LC-MS, which allowed detection of 1035 mass features (representing individual metabolites), of which 929 (characterized by <20% zero values) were included for biomarker discovery. Changes in plasma metabolite levels were characterized across 40 hours of sleep deprivation to show comparability across experiment 1 [*n* = 12 participants, 218 samples (10 samples were not collected due to cannulation or protocol issues)] and experiment 2 [*n* = 11 participants, 198 samples (11 samples were not collected)] (see the next section). Variable reduction was conducted on experiment 1 (training set) before random forest models were built (see the “Development: Selecting metabolite candidates for a biomarker of sleep deprivation” section) and then tested on experiment 2 (test set) using holdout analyses (see the “Validation: Predicting sleep deprivation using five final biomarker candidates (in an independent test set)” section). Further validation of biomarker response to sleep was assessed in a comparable well-rested (WR) protocol, with an 8-hour sleep opportunity overnight (matched control: 5 participants, *n* = 30). See fig. S1 for graphical representation of analysis plan.

### Characterization: Metabolomic changes at the group and individual level

#### Sleep deprivation experiment 1

**Fig. 1.**
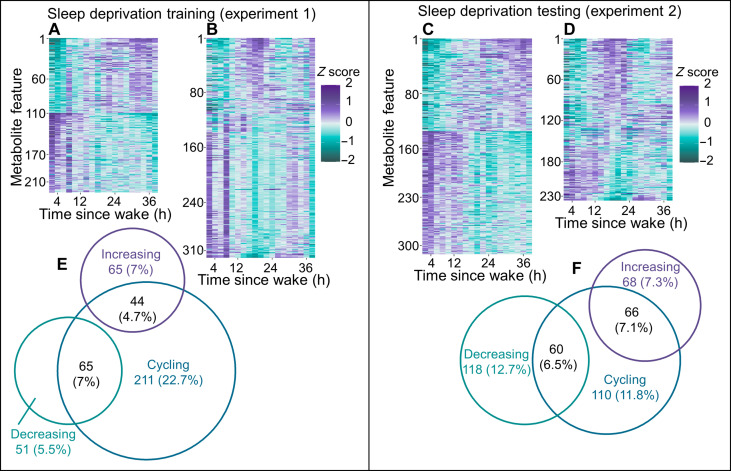
Features isolated by HILIC LC-MS and showing linear and/or cyclical trends across each sleep deprivation experiment. Heatmaps display significant (FDR-adjusted *P* value of <0.05) group-level trends during sleep deprivation for linear (**A**) and cycling (**B**) trends for experiment 1 (*n* = 12) and linear (**D**) and cycling (**E**) trends for experiment 2 (*n* = 11). For all heatmaps, purple corresponds to the highest and green corresponds to the lowest values for *z*-scored median-normalized peak area. Venn diagrams display the number and overlap of cycling, increasing or decreasing features for experiment 1 (**C**) and experiment 2 (**F**). The Venn ring sizes correspond to the number of features, and percentages are relative to the total number of features analyzed (929).

At the group level, and across 40 hours of sleep deprivation under CR conditions, 225 (24.2%) mass features displayed significant linear trends and 320 (34.4%) displayed significant cycling (amplitude of cosinor fit) trends, after adjusting for multiple comparisons [false discovery rate (FDR)]. Of the 320 cycling features, 44 (4.7%) increased and 65 (7%) decreased with TSW (Fig. 1, A to C ).

At the individual level, 30 (3.2%) mass features were significantly linear (with consistent increasing/decreasing trend across participants) and 10 (1%) were significantly cycling in more than 50% of participants. No mass features were both cycling and linear in more than 50% of participants. Volcano plots presenting the magnitude and significance of (individual and group) linear and cycling trends from both experiments, relative to all other mass features, are displayed in fig. S2.

#### Sleep deprivation experiment 2

Group-level linear and cycling trends were different between experiments, with experiment 1 presenting more cycling features and experiment 2 presenting more decreasing linear mass features (χ^2^ = 63.2, *P* < 0.001). Experiment 2 presented 312 (33.6%) mass features with significant linear trends and 236 (25.4%) that displayed significant cycling trends. Of the 236 cycling features, 66 (7.1%) increased and 60 (6.5%) decreased with increasing TSW (Fig. 1, D to F ).

At the individual level, the experiments were comparable (χ^2^ = 1.05, *P* = 0.590), with experiment 2 presenting 37 (4%) mass features that were significantly linear (with consistent increasing/trend across participants), and 16 (1.7%) that displayed significantly cycling trends in more than 50% of participants.

### Development: Selecting metabolite candidates for a biomarker of sleep deprivation

#### Feature selection

Mass features were selected for predictive modeling of sleep deprivation using a knowledge-based a priori approach followed by variable selection using random forest (VSURF). This approach allowed variable reduction (e.g., fewer metabolites entered into the model) while retaining all mass feature information so that appropriate candidates could be identified and assessed individually. Variable selection was conducted on data from experiment 1. Mass features that were linear in >50% of participants in the training experiment were selected (see Materials and Methods). Any mass features that presented a 24-hour rhythm (i.e., were cyclical) in more than 25% of participants were excluded from modeling to remove possible time of day confounds from the final sleep deprivation biomarker. Of the 929 features, 20 mass features (1.9%) passed this knowledge-based a priori feature selection and were therefore used in random forest modeling to create the biomarker of sleep deprivation.

#### Identification of filtered features (metabolites)

The 20 features included in predictive modeling were identified (where possible) on the basis of mass/charge ratio (*m*/*z*), retention time (RT), fragmentation patterns, and chemical standards (table S1). Six features (plus one isotope) were identified to level 1 using chemical standards. Four features were putatively identified to a single metabolite using online databases and fragmentation patterns (level 2). A further eight features (plus one isotope) were putatively identified to class (level 3), including monosaccharides and several lipid groups (table S1).

#### Final metabolite candidates selected for a biomarker of sleep deprivation

**Fig. 2.**
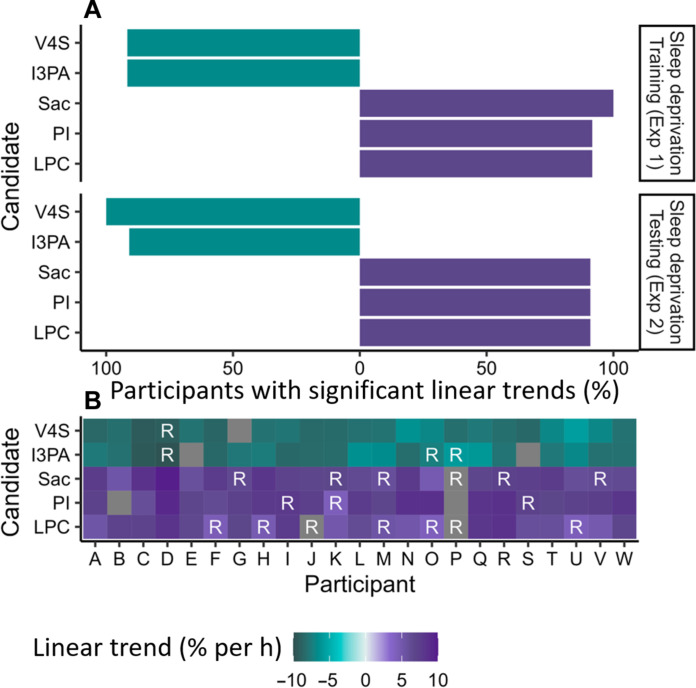
Significant linear changes at the individual level for each of the five final biomarker candidates across both sleep deprivation experiments. (**A**) Horizontal bar charts summarize the percent of participants displaying an increasing or decreasing trend in *z*-scored median-normalized peak area for each metabolite for sleep deprivation training set (experiment 1) and testing set (experiment 2). (**B**) Heatmaps display significant linear trends observed for each participant with increasing TSW for both experiment 1 (participants A to L) and experiment 2 (participants M to W). Linear trends are displayed as percent change per hour TSW, with decreasing trends in green and increasing trends in purple. Non-significant metabolite/participants are displayed in dark gray. Metabolites that were also significantly rhythmic are indicated with a white “R.”

Our metabolomic biomarker of sleep deprivation was developed to address different future applications (e.g., repeated testing in the same individual and single time-point testing). We therefore took a within- and between-participant approach to predictive modeling and built classification and regression models for each. Classification random forest models were initially built to classify WR versus clock time–matched sleep deprivation (SD) conditions [i.e., 0 to 16 hours compared to 24 to 38 hours awake (WR-SD)]. Regression random forest models were used to predict TSW. A reduced number of variables capable of explaining observed variance in TSW were selected from the 20 filtered features using VSURF. Minimizing the number of variables is preferential for biomarker implementation, where the development of standards and/or biosensors is time consuming and expensive. VSURF analyses selected between 6 and 14 variables as important for predicting sleep deprivation across the four analytical approaches (classification, regression, and within and between participant) in experiment 1 (table S2). Across the majority of VSURF models, five variables (features) were consistently selected as important at the interpretation level and thus formed the candidates for our biomarker of sleep deprivation (of note, these final five biomarker models performed equivalently to models using the VSURF selected and initial 20 filtered features; see table S2). The five final biomarker candidates were identified as vanillin 4-sulfate (level 1), indole-3-propionate (level 1), monosaccharide 1 (level 3), the phosphatidylinositol molecular species, PI(16:0/18:1) (level 1), and the lysophosphatidylcholine molecular species, LPC(18:3) (level 1) (Table 1). Individual trends observed for the five final biomarker candidates during the two sleep deprivation experiments are summarized in Fig. 2. Raw individual trends are shown in figs. S3 to S6, and the magnitude of group-level changes is shown in fig. S7. Linear trends were significant in almost all participants (>90%) for all five final biomarker candidates in both sleep deprivation experiments (Fig. 2, A and B ).

**Table 1.** Metabolite identification of the five candidates consistently selected by VSURF, including metabolite ID, *m*/*z*, RT, calculated formulas, ppm difference between formula and candidate *m*/*z*, and level of identification reached as described by the metabolite standards initiative. Metabolites in bold were confirmed to level 1 identification using standards.

Metabolite ID	*m*/*z*	RT (min)	Formula	ppm	Level
**Vanillin 4-sulfate**	**230.9955**	**4.0**	**C8H8SO6**	**−5.9**	**1**
**Indole 3-propionate**	**188.0722**	**7.3**	**C11H11NO2**	**2.5**	**1**
Monosaccharide 1	179.0561	13.3	C6H12O6	−0.3	3
**PI(16:0/18:1)**	**835.5352**	**3.3**	**C43H81O13P**	**1.2**	**1**
**LPC(18:3) + CH2O3**	**578.3101**	**4.1**	**C27H50NO10P**	**0.3**	**1**

### Validation: Predicting sleep deprivation using five final biomarker candidates (in an independent test set)

Following the development of the sleep deprivation biomarker above, we then validated the biomarker in an independent test set using unseen data.

#### Predicting sleep deprivation (24 hours awake) relative to WR conditions using five final biomarker candidates (within participant)

Median-transformed *z*-scored data (metabolite intensities were median-transformed within sample and autoscaled within participant) were used to assess within-participant changes. These analyses are applicable to situations where multiple samples can be taken from the same individual over time (e.g., continuous monitoring). The presence of sleep deprivation >24 hours (WR-SD) was predicted using classification random forest models. This approach is applicable in situations where detecting a threshold of time awake (i.e., 24 hours or more) is required, such as detection of prohibited or unsafe levels of prior sleep deprivation.

**Fig. 3.**
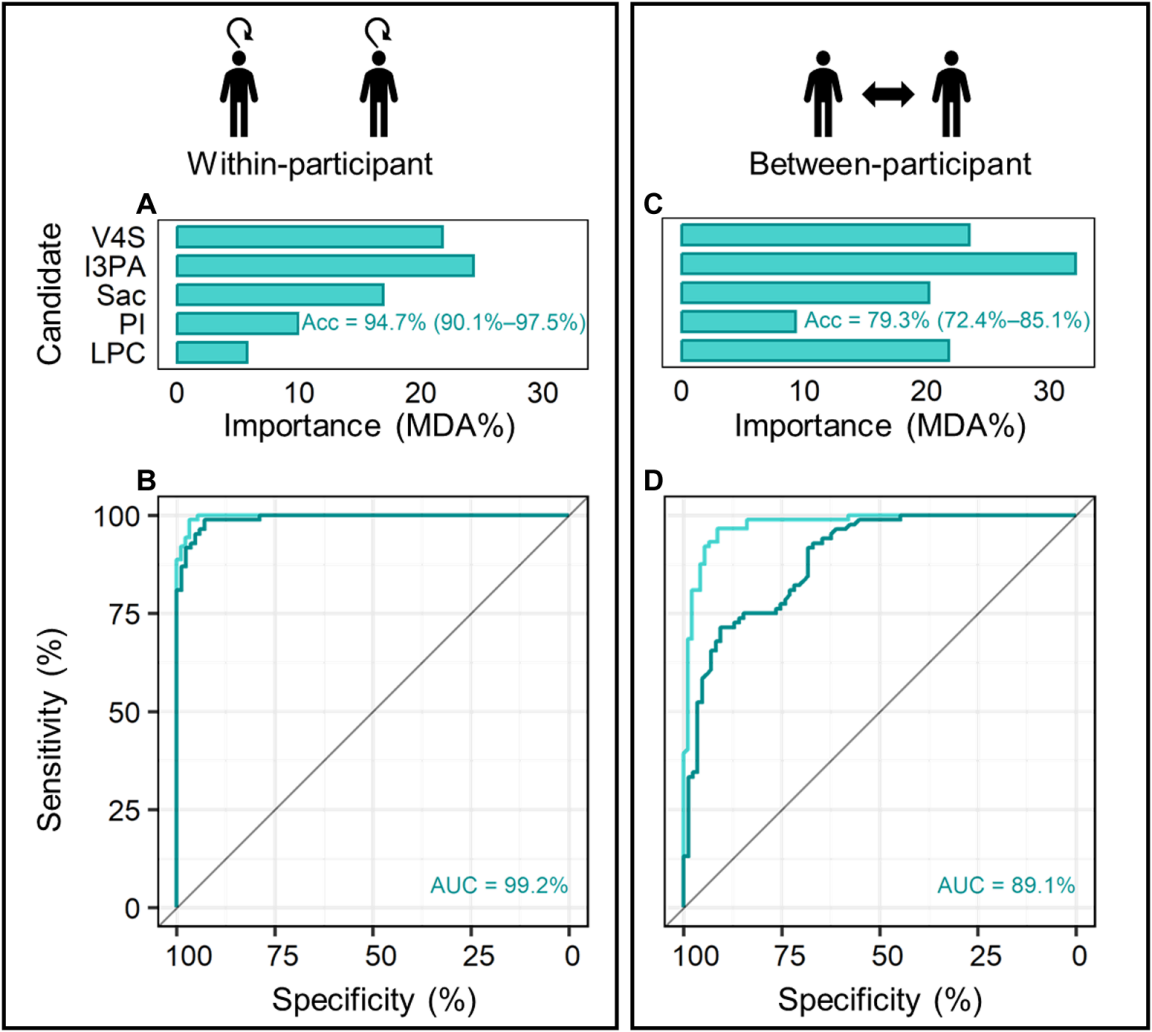
Classification random forest model results for within- and between-participant analyses. Top: Relative importance of the five final biomarker candidates for training models using within-participant (**A**) and between-participant (**C**) data. Testing model accuracies (lower to upper 95% CI) are displayed in each panel. Bottom: Receiver operating characteristic curves for within-participant (**B**) and for between-participant data (**D**). Training models are displayed in light green, and test models are in dark green. Acc, accuracy; AUC, area under the curve; MDA%, mean decreased accuracy on variable removal; V4S, vanillin 4-sulfate; I3PA, indole-3-propionate; Sac, monosaccharide 1; PI, PI(16:0/18:1); LPC, LPC(18:3).

The relative importance of the five final biomarker candidates for a classification model predicting WR-SD conditions is displayed in Fig. 3A. This model presented an overall training accuracy [lower to upper 95% confidence interval (CI)] of 97.8% (94.5 to 99.4) and a testing accuracy of 94.7% (90.1 to 97.5), correctly predicting 79 of 85 WR samples and 81 of 84 SD samples in test data. A negative prediction accuracy (NPV) of 92.9%, positive prediction accuracy (PPV) of 96.4%, specificity (SP) of 96.3%, and sensitivity (SN) of 93.1% were calculated from this model (Table 2). The receiver operator characteristic (ROC) curve of this model presented an area under the curve (AUC) of 99.2% (98.3 to 100) (Fig. 3B).

**Table 2.** Group- and individual-level results of classification [predicting clock time–matched >24 hours awake (0 to 16 hours to 24 to 38 hours awake)] and regression (predicting TSW) random forest models for within-participant and between-participant analyses. Showing models trained on experiment 1 (tested experiment 2 group and participants) using the five final biomarker candidates. For classification models, model accuracy (lower–upper 95% CI), area under the receiver operating curve (AUC %, lower–upper 95% CI), negative and positive prediction accuracy (NPV and PPV%), specificity (SP%), and sensitivity (SN%) are displayed (tree depth was 35 and 53, for within-participant and between-participant classification training models, respectively). Note that if PPV is 0, SN cannot be calculated. For regression models, variance explained (as *R*^2^) and RMSE are displayed (tree depth was 159 and 171, for within-participant and between-participant regression training models, respectively).

Comparison	Model	Participant	Accuracy (%) (lower–upper)	AUC (%) (lower–upper)	SP (%)	SN (%)	NPV (%)	PPV (%)	*R*^2^ (%)	RMSE
Within participant	Training	Group	97.8 (94.5–99.4)	99.7 (99.3–100)	98.9	96.7	96.8	98.9	98.0	1.52
	Testing	Group	94.7 (90.1–97.5)	99.2 (98.3–100)	96.3	93.1	92.9	96.4	86.7	4.06
		M	100 (79.4–100)	100 (100–100)	100	100	100	100	90.1	3.45
		N	93.8 (69.8–99.8)	100 (100–100)	88.9	100	100	87.5	89.4	3.57
		O	93.8 (69.8–99.8)	95.3 (85.1–100)	88.9	100	100	87.5	86.1	4.09
		P	81.2 (54.4–96)	95.3 (86.2–100)	100	72.7	62.5	100	57.7	7.13
		Q	93.8 (69.8–99.8)	100 (100–100)	88.9	100	100	87.5	93.4	2.90
		R	100 (79.4–100)	100 (100–100)	100	100	100	100	93.6	2.77
		S	100 (79.4–100)	100 (100–100)	100	100	100	100	84.6	4.30
		T	100 (79.4–100)	100 (100–100)	100	100	100	100	85.3	4.20
		U	92.9 (66.1–99.8)	100 (100–100)	100	87.5	85.7	100	87.4	4.49
		V	92.9 (66.1–99.8)	100 (100–100)	100	87.5	85.7	100	90.5	3.33
		W	92.3 (64–99.8)	100 (100–100)	100	85.7	85.7	100	93.5	2.84
Between participant	Training	Group	92.9 (88.1–96.1)	97.7 (95.8–99.6)	94.4	91.3	91.4	94.4	94.0	2.66
	Testing	Group	79.3 (72.4–85.1)	89.1 (84.4–93.8)	76.0	83.6	85.9	72.6	48.2	8.00
		M	75 (47.6–92.7)	100 (100–100)	66.7	100	100	50.0	51.9	7.60
		N	100 (79.4–100)	100 (100–100)	100	100	100	100	74.6	5.52
		O	68.8 (41.3–89)	90.6 (75.6–100)	100	61.5	37.5	100	28.6	9.25
		P	50 (24.7–75.3)	96.9 (89.6–100)	50.0		100	0.0	−28.4	12.41
		Q	87.5 (61.7–98.4)	100 (100–100)	100	80.0	75.0	100	65.0	6.65
		R	100 (79.4–100)	100 (100–100)	100	100	100	100	88.9	3.64
		S	50 (24.7–75.3)	94.5 (84–100)	50.0		100	0.0	−9.3	11.45
		T	81.2 (54.4–96)	100 (100–100)	72.7	100	100	62.5	56.5	7.23
		U	100 (76.8–100)	100 (100–100)	100	100	100	100	66.0	7.37
		V	85.7 (57.2–98.2)	90.8 (73.7–100)	100	77.8	71.4	100	69.1	6.00
		W	84.6 (54.6–98.1)	95.2 (84.2–100)	100	75.0	71.4	100	65.3	6.59

To examine performance at the individual level, models were further tested on each participant from the testing data (*n* = 19 time points per participant). As seen in Table 2, overall accuracy for the detection of SD was ≥92.3% in all but one participant (participant P: 81.2%). NPV was ≥85.7% in all but one participant (participant P: 62.5%), PPV and SP were ≥87.5% in all participants, and SN was ≥80% in all but one participant (participant P: 72.7%). While AUC for all individuals was ≥95.3%, this should be regarded with caution as the test datasets are small and AUC can be optimistic. Sanity checks presented similar results (table S3).

##### Reducing the distinction between WR and sleep deprivation conditions

After demonstrating the capacity to detect SD when classified as being awake for >24 hours (relative to a time-matched WR control of time awake < 16 hours), we then examined how model accuracy changed when using different thresholds of SD and WR. Classification models were rebuilt with stepwise inclusion of 18- to 22-hour TSW assessments in the WR (0 to 16 hours) or SD (24 to 38 hours) classifications. For within-participant analyses, these stepwise inclusions lowered model accuracy in a predictable way (Table 3), with test model accuracy dropping to a minimum of 90.4% (85.4 to 94.1) when comparing WR (0 to 22 hours) to SD (24 to 38 hours) TSW. Sanity check data are shown in table S4.

**Table 3.** Testing model results from classification random forests for within- and between-participant analyses for varying levels of sleep deprivation and WR classifications. Model accuracy (lower to upper 95% CI), area under the receiver operating curve (AUC, lower to upper 95% CI), negative and positive prediction accuracy (NPV and PPV%), specificity (SP%), and sensitivity (SN%) are displayed.

X	Time grouping (hours)	Accuracy (%) (lower–upper)	AUC (%) (lower–upper)	SP (%)	SN (%)	NPV (%)	PPV (%)
Within participant	0–16 vs. 18–38	92.4 (87.8–95.7)	98 (96.6–99.4)	91.7	93.0	90.6	93.8
	0–16 vs. 20–38	93.6 (89.1–96.7)	98.9 (98.1–99.8)	92.9	94.2	92.9	94.2
	0–16 vs. 22–38	93.8 (89.2–96.9)	98.8 (97.9–99.8)	93.0	94.6	94.1	93.5
	0–18 vs. 24–38	96.6 (92.8–98.8)	99.1 (98.1–100)	98.9	94.3	94.7	98.8
	0–20 vs. 24–38	92.1 (87.2–95.5)	97.9 (96.3–99.5)	95.0	88.8	90.5	94.0
	0–23 vs. 24–38	90.4 (85.4–94.1)	96.5 (94.2–98.7)	90.6	90.1	93.0	86.9
Between participants	0–16 vs. 18–38	70.2 (63.3–76.5)	84.7 (79.4–90)	64.8	74.5	67.1	72.6
	0–16 vs. 20–38	72.3 (65.4–78.6)	85.8 (80.6–91)	67.7	76.8	74.1	70.9
	0–16 vs. 22–38	75.3 (68.3–81.4)	87.6 (82.7–92.5)	71.1	80.2	81.2	69.9
	0–18 vs. 24–38	78.2 (71.4–84)	88.4 (83.7–93.1)	76.9	80.0	84.2	71.4
	0–20 vs. 24–38	77.2 (70.6–83)	88.1 (83.5–92.7)	78.7	75.3	81.0	72.6
	0–23 vs. 24–38	76.8 (70.3–82.5)	87.4 (82.8–92.1)	79.3	73.2	80.7	71.4

Note: For training models tested above, tree depth ranged from 27 to 73 nodes

##### Considering a lower level of sleep loss

As we tested biomarker performance against extensive sleep deprivation above (e.g., up to 38 hours within the classification bin), we then assessed the accuracy of predicting a lower level of sleep loss. We compared WR (0 to 16 hours) condition versus hours only in the biological night (18 to 24 hours) as the SD condition as this is more typical of the real world. Here, within-participant analyses resulted in a minimum test model accuracy of 84.6% (76.9 to 90.4) [NPV: 88.2%, PPV: 76.3%, SP: 89.3%, SN: 74.4%, and AUC: 95.1% (91.8 to 98.4)]. Widening the distinction to 20- to 24-, 22- to 24-, or 24-hours SD improved classification accuracy in a predictable way (table S5). As expected, the PPV decreased when comparing SD condition with low *n* (e.g., 24 hours only) relative to WR (versus 0 to 16 hours) as the models were unbalanced.

#### Predicting sleep deprivation (24 hours awake) relative to WR conditions using five final biomarker candidates for single-test samples (between participant)

Between-participant analyses using median-transformed data were used to assess trends across participants without removing between-participant variation. These analyses are applicable to situations requiring a single time-point sample (e.g., roadside or post-accident testing of time awake). For between-participant data, the relative importance of the five final biomarker candidates in a classification random forest model to predict the difference between WR-SD conditions is presented in Fig. 3C. This model presented an overall training accuracy of 92.9% (88.1 to 96.1) and a testing accuracy of 79.3% (72.4 to 85.1), correctly predicting 73 of 85 WR samples and 61 of 84 SD samples in the unseen test data. The model presented a testing NPV of 85.9%, PPV of 72.6%, SP of 76.0%, and SN of 83.6% (Table 2). The testing ROC curve produced from this model presented an AUC of 89.1% (84.4 to 93.8)Fig. 3D). This model displayed equivalent performance to models including the initial 14 VSURF selected variables [AUC = 90.1% (85.6 to 94.6)] and all 20 filtered features [AUC = 88.4% (83.5 to 93.4); table S2]. At the individual level, between-participant classification models predicted with lower accuracy than within-participant data, presenting an overall accuracy of ≥75% in 7 of 11 participants with a minimum of 50% in 2 participants (participants P and S) (Table 2). In both these participants, all samples were predicted to be WR (false negative, PPV: 0% and NPV: 100%). Sanity checks displayed similar results, although participant E was predicted to be SD when they were WR (false positive, PPV: 100% and NPV: 0%) (table S3).

##### Closing the distinction between WR and sleep deprivation conditions

Closing the distinction between WR and SD by stepwise inclusion of 18- to 22-hour TSW measurements in each condition for between-participant analyses resulted in a minimum test model accuracy of 70.2% (63.3 to 76.5) (NPV: 67.1%, PPV: 72.6%, SP: 64.8%, SN: 74.5%, and AUC: 84.7%), when comparing 0 to 16 hours as WR versus 18 to 38 hours awake as SD (Table 3). When comparing below/above the 24-hour threshold, the testing accuracy was 76.8% (63.3 to 76.5) (NPV: 80.7%, PPV: 71.4%, SP: 79.3%, SN: 73.2%, and AUC: 87.4%).

##### Considering a lower level of sleep loss

Comparing WR (0 to 16 hours) with hours only in the biological night (18 to 24 hours) as the SD condition, between-participant analyses resulted in a minimum test model accuracy of 69.1% (60.1 to 77.1) (NPV: 71.8%, PPV: 63.2%, SP: 81.3%, SN: 50.0%, and AUC: 76.9%), when comparing 0 to 16 hours as WR versus 18 to 24 hours awake as SD. Widening the distinction to 20 to 24 hours, 22 to 24 hours, or 24 hours SD improved classification accuracy in a predictable way (table S5).

#### Predicting TSW using the five final biomarker candidates

Increasing TSW was predicted using regression random forest models. This approach may be applicable in fitness-for-duty test scenarios where a number of hours awake at the start of the shift could result in excessive time awake during the course of a shift (e.g., long-haul drivers and pilots) and where variability in test timing precludes a single cutoff (classification). Linear trends for the five final biomarker candidates are displayed in the Supplementary Materials for within-participant analyses (figs. S3 and S4) and between-participant analyses (figs. S5 and S6).

##### Within-participant assessment for repeated testing/monitoring

**Fig. 4.**
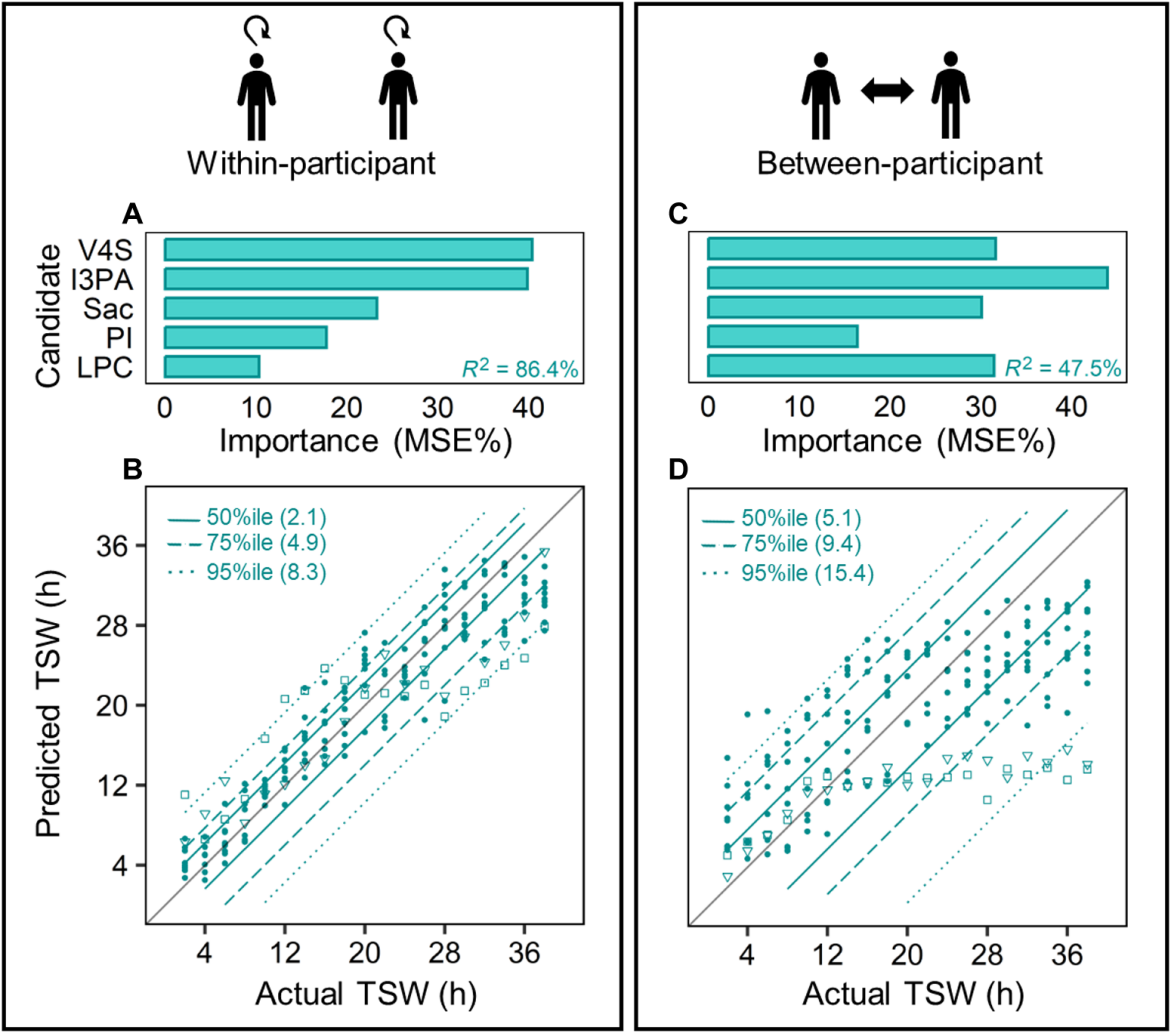
Random forest model regression results for within- and between-participant analyses. Top: Relative importance of the five final biomarker candidates for training models using within-participant (**A**) and between-participant data (**C**). Testing *R*^2^ is shown in each panel. Bottom: Model predicted TSW versus actual TSW in test data for within-participant (**B**) and between-participant models (**D**). Line types indicate percentiles with corresponding prediction error shown in hours (e.g., 50% of individual points are predicted within 2.1 or 5.1 hours of the actual time awake for within- and between-participant, respectively). Two outlier participants (P = □; S = ▽) are highlighted (adjusted *R*^2^ for between-participant is 63.8% with their removal). MSE%, increase in mean square error on variable removal; TSW, time since wake.

The relative importance of the five final biomarker candidates for a regression random forest predicting within-participant changes with increasing TSW is displayed in Fig. 4A. This model presented a training *R*^2^ of 98.1% [root mean square error (RMSE) of 1.51 hours] and a testing *R*^2^ of 86.4% (RMSE of 4.14 hours) (Fig. 4A and Table 2). The model predicted 50% of data ± 2.1 hours of the actual value (Fig. 4B). To examine model performance at the individual level, these models were further tested on each individual (*n* = 19 time points per participant). This presented *R*^2^ > 80% and RMSE < 4.6 hours in 10 of 12 participants, with median *R*^2^ = 90.3% and RMSE = 3.41 hours. The maximum *R*^2^ was 93.8% (RMSE 2.78 hours) in participant W, and the minimum *R*^2^ was 56.2% (RMSE 7.25 hours) in participant P (Table 2). Sanity checks presented comparable results (table S3).

##### Between-participant assessment for single-test sample detection

The relative importance of the five final biomarker candidates for regression random forest predicting between-participant changes with increasing TSW is displayed in Fig. 4C. This model presented a between-participant training *R*^2^ of 94.1% (RMSE of 2.64 hours) and a testing *R*^2^ of 47.8% (RMSE of 8.03 hours) (Table 2). The model predicted 50% of data ± 5.1 hours of the actual value (Fig. 4D). At the individual level in the testing data, between-participant regression models explained above 80% (*R*^2^) variation in only 1 of 11 participants, >50% *R*^2^ was achieved in 8 of 11 participants, and in two participants *R*^2^ was <0% (Table 2). Median *R*^2^ and RMSE were 63.0% and 6.64 hours, respectively.

#### Translational utility—Predicting sleep deprivation with fewer metabolites

As we were unable to identify one of the five final biomarker candidates (monosaccharide 1), replicability of this biomarker is affected. For biosensor/device development, having fewer candidates is preferable if prediction accuracy remains unchanged. We therefore modeled all possible combinations of two or more of the five final biomarker candidates (26 combinations) for our initial comparison of WR (0 to 16 hours) with time-matched SD (24 to 38 hours). Although the monosaccharide was of medium importance, its removal reduced accuracy only slightly for the classification (2.4 and 3.6% reduction) and regression (3.6 and 7.4% reduction) models for within- and between-participant analyses, respectively. Outputs from all models are summarized in table S6.

#### Biomarker recovery with sleep

**Fig. 5.**
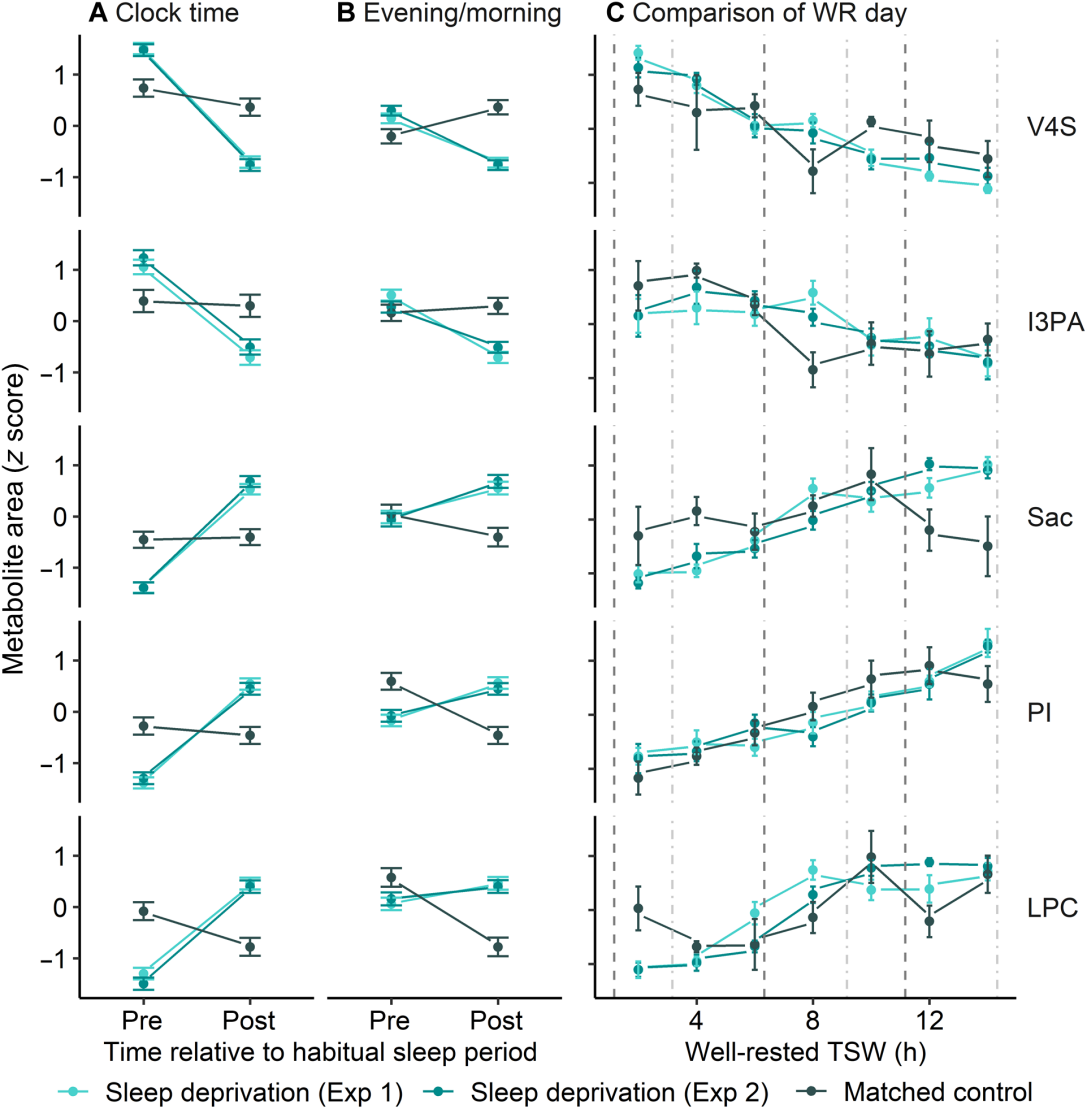
Group-level trends of the five final biomarker candidates in sleep deprivation and matched control experiments. (**A** and **B**) Mean candidate metabolite levels (*z*-score) from a 4-hour block pre- and post-habitual sleep interval were compared between sleep deprivation and matched control protocols. Pre- and post-habitual sleep were compared using both clock time–matched (A) (sleep deprivation—pre: 2 to 6 hours TSW/post: 26 to 30 hours TSW; matched control pre: 2 to 6 TSW day 1/post: 2 to 6 TSW day 2) and (B) evening/morning (sleep deprivation—pre: 12 to 16 hours TSW/post: 26 to 30 hours TSW; matched control pre: 12 to 16 hours TSW day 1/post: 2 to 6 hours TSW day 2). (**C**) A comparison of a WR day 3 from sleep deprivation (CR) and matched control (CP) protocols. Dashed lines indicate main meals (dark gray) and snacks (light gray) in the matched control protocol. Snacks were provided hourly in the sleep deprivation protocols. Series colors indicate the protocol [sleep deprivation (experiment 1): light green, sleep deprivation (experiment 2): dark green, and matched control: charcoal].

The final step in validation for our sleep deprivation biomarker was to examine the recovery of these biomarkers following a sleep interval. This was investigated using a matched control experiment, where participants completed a protocol comparable to the sleep deprivation experiment [e.g., constant posture (CP) and dim light conditions, although without regular hourly meals] but where an 8-hour sleep opportunity was provided at their habitual sleep time, resulting in a typical 16-hour:8-hour wake/sleep schedule. At the group level, the five final biomarker candidates displayed opposite (or nonsignificant trends) before and after the sleep interval compared with those observed between the same clock times in the sleep-deprived groups. This effect was consistent when comparing before and after sleep using clock time–matched groups (e.g., 24 hours apart) of 2 to 6 hours versus 26 to 30 hours TSW in the sleep deprivation experiment with 2 to 6 hours TSW each day for the matched control (Fig. 5A) and the evening/morning before/after sleep times (e.g., 12 to 16 hours versus 26 to 30 hours TSW in the sleep deprivation experiment and 12 to 16 hours day 1 versus 2 to 6 hours day 2 for matched control) (Table 4 and Fig. 5B). At the individual level, opposite or nonsignificant trends relative to that observed during sleep deprivation were observed in most matched control participants (fig. S6 and table S7). A single exception was participant 4, who displayed a clock time–matched decrease in vanillin 4-sulfate, although their evening/morning comparison was not significant (FDR-adjusted) (fig. S8 and table S7).

**Table 4.** Mixed linear models comparing candidates pre- and post-habitual sleep compared to time points from the sleep deprivation experiments. Candidate *z*-scores during a 4-hour block pre- and post-habitual sleep interval for clock time–matched (2 to 6 hours versus 26 to 30 hours) and evening/morning (12 to 16 hours versus 26 to 30 hours) in two sleep deprivation and the matched control experiments. Results are displayed as estimate, degrees of freedom (df), *t* ratio, *P* value, and the trend observed between pre- and post-means.

Time comparison	Metabolite	Protocol	Estimate	df	*t* ratio	*P* value (FDR-adjusted)	Trend
Clock time–matched	Vanillin 4-sulfate	Sleep deprivation (experiment 1)	−2.2	134.7	−14.3	<0.001	Decreasing
		Sleep deprivation (experiment 2)	−2.2	134.1	−13.7	<0.001	Decreasing
		Matched control	−0.4	134.1	−1.6	0.143	Not significant
	Indole 3-propionate	Sleep deprivation (experiment 1)	−1.8	134.3	−11.4	<0.001	Decreasing
		Sleep deprivation (experiment 2)	−1.7	134.0	−10.6	<0.001	Decreasing
		Matched control	−0.1	134.0	−0.4	0.721	Not significant
	Monosaccharide 1	Sleep deprivation (experiment 1)	1.9	134.7	13.3	<0.001	Increasing
		Sleep deprivation (experiment 2)	2.1	134.1	13.7	<0.001	Increasing
		Matched control	0.0	134.1	0.2	0.824	Not significant
	PI(16:0/18:1)	Sleep deprivation (experiment 1)	1.9	134.7	12.5	<0.001	Increasing
		Sleep deprivation (experiment 2)	1.7	134.1	10.8	<0.001	Increasing
		Matched control	−0.2	134.1	−0.8	0.49	Not significant
	LPC(18:3) + CH_2_O_3_	Sleep deprivation (experiment 1)	1.8	134.7	10.9	<0.001	Increasing
		Sleep deprivation (experiment 2)	1.9	134.1	11.1	<0.001	Increasing
		Matched control	−0.7	134.1	−2.8	0.008	Decreasing
Evening/morning	Vanillin 4-sulfate	Sleep deprivation (experiment 1)	−0.9	128.7	−6.5	<0.001	Decreasing
		Sleep deprivation (experiment 2)	−1.1	130.1	−7.8	<0.001	Decreasing
		Matched control	0.6	128.1	2.9	0.007	Increasing
	Indole 3-propionate	Sleep deprivation (experiment 1)	−1.2	128.6	−8.4	<0.001	Decreasing
		Sleep deprivation (experiment 2)	−0.8	129.8	−5.4	<0.001	Decreasing
		Matched control	0.1	128.1	0.6	0.564	Not significant
	Monosaccharide 1	Sleep deprivation (experiment 1)	0.6	128.6	3.5	0.002	Increasing
		Sleep deprivation (experiment 2)	0.8	129.7	4.5	<0.001	Increasing
		Matched control	−0.5	128.1	−1.9	0.076	Not significant
	PI(16:0/18:1)	Sleep deprivation (experiment 1)	0.7	128.7	4.6	<0.001	Increasing
		Sleep deprivation (experiment 2)	0.5	130.1	3.3	0.002	Increasing
		Matched control	−1.1	128.1	−4.5	<0.001	Decreasing
	LPC(18:3) + CH_2_O_3_	Sleep deprivation (experiment 1)	0.4	128.7	2.3	0.029	Increasing
		Sleep deprivation (experiment 2)	0.2	130.1	1.4	0.203	Not significant
		Matched control	−1.4	128.1	−5.3	<0.001	Decreasing

To investigate the influence of meal timing on biomarker candidate levels, trends of data *z*-scored across WR days from the matched control (CP, with three large meals and three snacks) and sleep deprivation (CR, with hourly snacks) protocols (day 3) were compared. A mixed linear model with TSW and study protocol (meal schedule) as fixed factors and participant as a random factor indicated that all metabolites altered with increasing TSW, but these trends were not significantly different between protocols/diet (Fig. 5C; *P* > 0.97).

## DISCUSSION

This study developed and validated a metabolomic biomarker that predicted >24 hours time awake and therefore the presence of acute sleep deprivation due to extended wake in young healthy adults. The metabolomic biomarker was tested for accuracy, replicability, and deployment potential. First, in an unseen test set, this biomarker was accurate in predicting the presence of sleep deprivation (>24 hours, 94.7% accuracy/AUC = 99.2%) and time awake (*R*^2^ = 86.1%) when compared to a WR sample. While accuracy remained very good to excellent for a single time-point detection of sleep deprivation (79.3% accuracy/AUC = 89.1%), it was lower when detecting increasing time awake (*R*^2^ = 45.6%). Second, replicability is supported by robust identification of individual metabolites, which comprised indole 3-propionate, vanillin 4-sulphate, PI(16:0/18:1), and LPC(18:3). Inclusion of a monosaccharide (currently unidentified) further increased accuracy. Third, we achieved this biomarker of sleep deprivation with only five metabolites, thus facilitating the development of standards, functionalized biosensors, and detection devices to promote future deployment to accurately and rapidly detect sleep deprivation in a point-of-care setting.

A biomarker of acute sleep deprivation must be able to identify sleep loss at the individual level, demonstrating high SN (correctly identifying those with insufficient sleep) and/or high SP (correctly identifying those with sufficient sleep). In an unseen test dataset, this biomarker predicted 24 hours or more of sleep deprivation with an accuracy of 94.7% (AUC = 99.2%), correctly identifying 81 of 84 sleep-deprived samples (SN = 93.1%) and 79 of 85 WR samples (SP = 96.3%). When moving toward a real-world application where the distinction between WR/sleep deprivation may be closer (i.e., samples <24 hours versus those >24 hours), accuracy remained high (90.4%, AUC = 96.5%). When detecting a lower level of sleep loss that may be typical in real-world environments (i.e., 0 to 16 versus 18 to 24 hours), accuracy dropped only slightly (84.6%, AUC = 95.1%). To our knowledge, this is the most accurate metabolomic biomarker of sleep loss described to date, although we respectfully acknowledge the difference in sleep loss schedules compared to prior studies. For example, the metabolomic prediction model of chronic sleep restriction previously described an accuracy of 74% (*27*). We also observe comparatively higher levels of accuracy (94.7% versus 74%) for predicting clock time–matched within-participant acute sleep deprivation, relative to previous studies using a transcriptomic approach (*22*). However, because of the difference in data processing used in our study and others (within-participant correction and machine learning approach), it is unclear whether the improvement is biological, methodological, or analytical in nature. Notwithstanding, this metabolomic biomarker has potential for future implementation in operational environments, where the cost of a sleep-related error is high (e.g., transportation or space exploration) and where WR samples can be obtained as either a prescreen or follow-up.

The prediction of between-participant sleep deprivation resulted in a lower accuracy (79%, AUC = 89.1%), relative to the within-participant approach, although we note still considered very good to excellent. This small reduction in accuracy is likely due to previously described interindividual variation in metabolomic profiles (*25*, *26*), whether biological or technical in nature. Despite this, and similar to the within-participant analyses, there remained only a marginal reduction in detection accuracy when the distinction between sleep deprivation and WR was reduced (e.g., <24 hours versus >24 hours; accuracy = 76.8%, AUC = 87.4%). Our biomarker performed similarly to a previous biomarker developed from whole-blood transcriptomics, which predicted <24 hours versus >24 hours in between-participant data with 80% accuracy (*22*) [although we note that in the transcriptomic study, a wider distinction between WR and SD (7 to 8 hours versus 31 to 32 hours TSW) achieved a 92% accuracy]. These biomarkers have important implications for translational pathways requiring a single test sample, such as roadside testing or post-accident analysis of suspected fall asleep crashes. Here, the potential improvements in road safety are considerable both directly, such as detecting drivers who may represent a substantial risk to themselves and others on the road, and indirectly through unmasking the true cost of sleep deprivation in road trauma (*29*). For safety-critical environments or medical diagnosis, SN is considered paramount, where detecting individuals who are impaired is preferred over the cost of a false positive. This is in contrast to the use of a biomarker as forensic evidence, where SP is considered more critical. In this latter example, a high proportion of false positives would jeopardize evidence credibility such that operational parameters are estimated to be at least 85% SP (true negative detection) (*30*). As this threshold exceeds that reported for our between-participant (single time point) biomarker (SP = 73.3%), several adjustments would need to be made, including (i) a second sample being obtained under WR conditions for any driver/worker suspected as “sleep-deprived,” (ii) optimization of the biomarker through a multisystems approach (e.g., with related biological measures, such as mRNA, proteins, or enzymes), or (iii) adjustment of the threshold to optimize SP at the cost of reduced SN. In this latter respect, adjusting our biomarker parameters to meet this requirement (SP = 85%) resulted in an SN of 65.5% when comparing above and below 24 hours awake (i.e., a closed distinction between pass/fail). This result suggests that the developed biomarker and adjusted threshold for minimizing false positives (to less than 15%) has the potential to detect 65.5% of drivers or operators who have been awake for 24 hours or longer and represent a serious danger to road safety (i.e., performance at this level is considered more detrimental than any legal limit of alcohol worldwide). Subject to validation in the wider population, and under less well-controlled environments, these data have important implications for jurisdictions where driving while knowingly fatigued/drowsy (e.g., being without sleep for a period in excess of 24 consecutive hours—Maggies Law, New Jersey) is a prosecutable offense.

Regression models of within-participant time awake predicted 50% of samples within 2.1 hours of actual TSW (*R*^2^ = 86.4%), although the between-participant models were weaker (*R*^2^ = 45.6%). While these correlations were stronger than those observed by Laing *et al.* (*22*) (*R*^2^ ~ 30% for within and between participants) possibly due to the nonlinear and nonparametric nature of random forest models, the percentage of variance explained for time awake for a single sample remains low. Several important observations can be made in this respect. First, our predicted TSW was generally lower than actual TSW [e.g., 38 hours TSW was predicted as 28 to 36 hours for within and 20 to 28 hours TSW (with outliers of 12 hours)], which may be due to a floor effect of the metabolite due to rapid depletion or reaching detection limits before excessive (>20 hours) sleep loss. This can be seen in Fig. 4 where the prediction model plateaus after 12 to 20 hours awake (and particularly evident for participants P and S). Second, these data should not be interpreted as the metabolites themselves being irrelevant for the detection of time awake (as confirmed with classification), but rather that the regression model may not be the most fruitful approach for future biomarker development due to these trends. For future studies where actual TSW is required, using targeted assays (where metabolites are known) in combination with new systems (e.g., high SN mass analyzers) or approaches (e.g., enrichment) may enhance detection of low abundant metabolites and improve performance of the regression approach. Notwithstanding, this sleep biomarker may have utility in TSW detection where repeat samples are available and no required time threshold exists. Furthermore, as several of these biomarkers have been implemented in health (see below) and our regression models are applicable to ongoing health monitoring, our work may be applicable to future sleep health wearables with continued monitoring of sleep health. With further research on the health implications of these metabolites, and the influence of sleep deprivation on their function, the health effects of insufficient sleep could be better understood and monitored. In addition, for individual differences such as those who present as WR during sleep deprivation (as observed in participants P and S), this type of monitoring may offer insight into individual resilience or vulnerability to the health impacts of sleep deprivation.

Beyond total sleep deprivation, partial or chronic sleep restriction is frequently experienced by those working night shifts (*31*), extended duration work shifts (*32*), those with sleep disorders (e.g., insomnia and sleep apnea), or for many who remain awake overnight for social and recreational or caregiving reasons (*33*). The extent to which our biomarker may detect those experiencing insufficient sleep due to sleep restriction/curtailment (e.g., 3-hour sleep) is unknown. Moreover, research suggests that metabolites representing sleep loss may differ to those representing sleep duration. For instance, to our knowledge, none of the five metabolites included in our biomarker of sleep deprivation were identified among the 65 metabolites suggested by Depner *et al.* (*27*) as important for the detection of insufficient sleep. Furthermore, this discrepancy between biomarkers reflecting sleep or lack thereof was also reported by Laing *et al.* (*22*) and further work exploring this is required. It also remains unknown how the biomarker developed here would respond to short sleep bouts or “naps,” which has implications for its potential future use, for example, in situations where individuals are encouraged to take a 20-min “power nap” to restore their alertness and ability to undertake safety-critical tasks (*34*). Although we demonstrate the recovery of all five metabolites following a night of sleep, future work should examine recovery over shorter time scales (e.g., 30 min, 1 hour, etc.) and/or develop complementary metabolites representing sleep duration and showing rapid recovery following sleep. Last, the behavioral response to sleep loss shows large interindividual variability such that, while one person may respond poorly to sleep loss (“vulnerable”), others may remain relatively intact (“resilient”) (*35*). As this biomarker was developed to detect a physiological response to sleep loss, future work is required to understand whether this biomarker predicts the behavioral vulnerability to sleep loss.

To aid replicability of this biomarker, we provide high-confidence identifications for four (of five) of these metabolites. Beyond providing a biomarker of sleep deprivation, these metabolites represent biological processes that are altered by sleep deprivation and thus provide information regarding the link between sleep deprivation and health. Vanillin 4-sulphate is a phase 2 enzyme-conjugated metabolite of the phenolic compound vanillin, predominantly formed in the kidneys or liver from the dietary precursor vanillin or in the gut from ferulic acid (*36*, *37*). A decrease in vanillin 4-sulphate may indicate reduced liver function or increased antioxidant/anti-inflammatory action of its precursors. This could be a potential mechanism associating poor sleep and liver disease (*38*). Indole-3-propionate is a gut bacteria–derived tryptophan metabolite, and one of the few antioxidants not to produce a pro-oxidant intermediate when scavenging hydroxyl radicals (similar to melatonin) (*39*, *40*). Indole-3-propionate is considered protective in several conditions for which oxidative damage is a concern (e.g., Alzheimer’s disease) (*41*, *42*) and metabolic disorders [e.g., type 2 diabetes; (*43*–*45*)]. These conditions are closely associated with sleep disturbance, with risk factors inversely related to sleep quality/quantity (*46*–*48*). Here, the decrease in indole-3-propionate could present a mechanism associating sleep deprivation and metabolic or age-related diseases. Last, the two remaining identified biomarker candidates were lipids, supporting findings that lipid metabolism is important in sleep and sleep deprivation (*24*, *25*, *28*, *49*–*51*). More specifically, PI(16:0/18:1) is a membrane phospholipid involved in membrane transport and cell signaling (*52*) whereby increased levels of PI(16:0/18:1) as seen here could be due to membrane breakdown due to oxidative damage. LPCs such as LPC 18:3 can act via G protein–coupled receptor signaling and can enhance inflammatory responses disrupting mitochondrial integrity or inducing apoptosis. While the optimal level of LPCs in plasma has not yet been established, altered levels of LPCs have been associated with risk factors for several diseases, including cardiovascular, diabetes, and Alzheimer’s disease (*53*).

There were a number of limitations to this study. First, the study cohort of healthy young adults comprised more men than women (18:5), and therefore, our results may not generalize to the wider population, including those who are older or have a clinical diagnosis (particularly those relating to sleep or the metabolites themselves) (*54*, *55*). Although we did not observe any sex differences in our data, female participants were studied during their follicular phase to minimize differences due to menstrual phase in their behavioral response to sleep deprivation (*56*). Second, we present data from a small number of individuals (model trained on *n* = 11 and tested on *n* = 12) with repeated samples. This approach of highly controlled protocols with deeply phenotyped participants is typical of early biomarker development studies (*22*, *57*) and a strength of the current study. Future work should expand to larger populations under less controlled conditions (*12*, *54*, *57*). Third, hourly iso-caloric meals in the CR may alter metabolism incrementally each hour (comparable to the linear trends attributed to increasing wakefulness), although our examination of the WR day 3 control protocol indicated that the final five biomarker candidates were not significantly altered by meal timings. However, introducing variation in light, exercise, temperature, and feed fast cycles will be another important validation step for this biomarker, particularly for those biomarker metabolites related to metabolism and energy production (*27*). Furthermore, how these metabolites and their response to time awake is affected by population differences, such as ethnicity, age, medical conditions, and diet, needs to be investigated further. Using a combination of metabolites (these and others) in future work, we can likely account for this variation, however, as a combination of markers will be more robust to individual differences than a single marker (*17*, *27*, *28*).

To summarize, through a rigorous, laboratory-based study, we developed a biomarker comprising five metabolites, which together predict >24 hours awake with high accuracy. Using multiple samples in a fitness-for-duty design (within participant), this biomarker predicted sleep deprivation with more than 90% accuracy. In a single-sample (between-participant) design applicable in random testing such as in post-accident analysis, >75% accuracy was achieved. Notwithstanding real-world confounds, using this single time-point biomarker with an adjusted threshold acceptable in forensic settings (<15% false positives), 65.5% of sleep-deprived individuals (>24 hours awake) would be detected. By minimizing the number of candidates, and confirming their identity, we increased the translational capacity of this biomarker for device development in the long-term and forensic examination where blood samples may be obtained in the short term (e.g., suspected fall asleep crash or critical event). A biomarker based on this research has the potential therefore to be transformative for community safety both directly in detecting sleep-deprived individuals and indirectly through deterrence from undertaking safety-critical tasks while sleep-deprived. We therefore present a major step forward in next-generation detection of sleep loss with potential to reduce road and workplace injuries and fatalities.

## MATERIALS AND METHODS

### Experimental design

A between- and within-participant design examining changes in metabolite profiles across three experiments comprised of 2 × 40-hour extended wakefulness under CR conditions (train and test datasets) and 1 × 40 hours under comparable conditions but including an 8-hour interval of sleep during habitual sleep timing.

### Participants

Young healthy participants with regular sleep schedules were recruited from the general public as described previously (*26*, *58*). Participants either followed the protocol of a sleep deprivation experiment [experiment 1: *n* = 12 (25.6 ± 3.9 years old, one female), experiment 2: *n* = 11 (25.2 ± 7.4 years old, four females)], or the matched control *[*n** = 5 (24 ± 2.5 years old, all male)], and LC-MS processing was conducted as three separate experiments.

For all experiments, participants were healthy, with no medical, psychiatric, or sleep disorders and no drug, supplement, nicotine, caffeine, or alcohol use reported for 3 weeks before, and until completion of, the in-laboratory study. Participants were also nonshift working adults and had not crossed time zones in the 3 months preceding the study. Women were admitted to the in-laboratory study during the follicular phase of their menstrual cycles to minimize differences due to menstrual phase. Urine screening (for drugs and pregnancy) was conducted before laboratory admission. Further detail on recruitment criteria has been published elsewhere (*26*, *58*). The study was approved by the Monash University Human Research Ethics Committee (CF14/2790-2014001546).

### Sleep deprivation study protocol

**Fig. 6.**
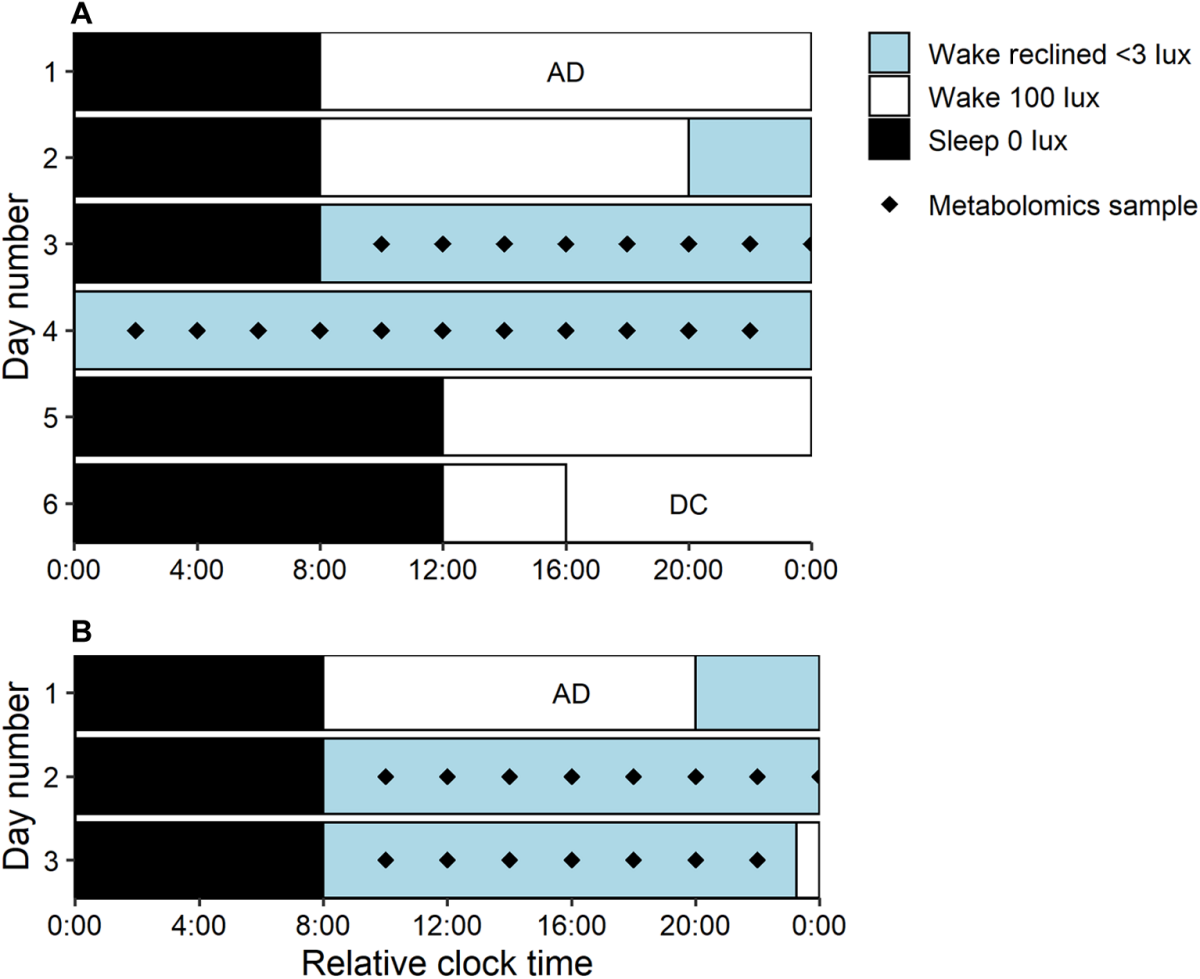
Raster plots depicting the in-laboratory protocols used in sleep deprivation experiments 1 and 2 and the matched control study. (**A**) The 6-day protocol used in sleep deprivation experiments. Days 1 and 2 were baseline days with 8-hour:16-hour sleep/wake cycle based on average habitual sleep before admission (AD). On day 3, the 40-hour CR commenced ending at the end of day 4. Days 5 and 6 were recovery days with up to 12-hour sleep opportunities before discharge (DC) on day 6. (**B**) The 3-day in-laboratory protocol used in the matched control study. Day 1 was the baseline day, and each day followed a self-selected 8-hour:16-hour sleep/wake cycle. Black diamonds represent blood samples taken for metabolomics analysis. White bars represent wake intervals in 100 lux, black bars represent sleep intervals in 0 lux, and gray bars represent wake intervals in <3 lux ambient light. The protocol is shown in relative clock time with a relative bedtime of midnight. Study events were scheduled relative to each individual’s pre-study self-selected wake time.

Sleep deprivation is defined here as a night of extended wake (acute sleep loss as opposed to restriction acute or chronic). For experiments 1 and 2, participants maintained a self-selected 8-hour:16-hour sleep/wake schedule for 2 weeks before the in-laboratory study [confirmed by sleep diaries and wrist actigraphy (Actiwatch Spectrum, MiniMitter Inc., Bend, OR)]. The sleep deprivation protocol is displayed in Fig. 6A. Participants were admitted to the laboratory and continuously monitored for 6 days in an environment free of time cues. The first two nights of the study were baseline sleep/wake, scheduled to each participant’s prelaboratory self-selected sleep schedule. Full polysomnography was recorded on the first night to confirm no presence of sleep disorders. During baseline days, participants were fed three main meals and three snacks per day, and lighting was 100 lux during wake and 0 lux during sleep (see the “Lighting conditions” section for more detail). On day 3, participants woke to start the 40-hour CR. Here, they remained awake under constant supervision in dim light conditions (<3 lux), in a semi-recumbent posture (head of bed at 45°). They consumed identical hourly snacks (quarter sandwich, 60 ml of water, and 40 ml of apple juice) with a calorie and macronutrient content (~20% protein, ~33% fat, and ~46% carbohydrate) in line with the Australian Dietary Guidelines (*59*).

### Matched control study protocol

For the matched control, each participant maintained a self-selected 8:16 sleep-wake schedule for 1 week before the in-laboratory stay. The matched control in-laboratory protocol was two nights (3 days) of 8-hour:16-hour sleep/wake schedule, scheduled to each participant’s prelaboratory self-selected sleep schedule (Fig. 6B). During wake intervals on days 2 and 3, participants underwent a constant posture (CP), where electroencephalography (EEG) measurements, lighting, posture, and biological sampling were matched to the CR protocol in experiments 1 and 2. Participants were fed three main meals and three snacks that were calorie-matched each day during the matched control, as it is impractical to feed hourly CR meals during a protocol with a sleep interval.

### Lighting conditions

During baseline and recovery days, maximum ambient light during wake episodes was ~102 ± 37 lux (horizontal plane) and ~45 ± 21 lux (vertical plane). Lights were dimmed to ~3 ± 1 lux (horizontal) and ~1 ± 3 lux (vertical) for the last 5 hours of wake on baseline night 2 for 6-day study (experiments 1 and 2), and during the CR (experiments 1 & 2), and CP wake episodes (days 2 and 3) of the matched control study (Fig. 6). During scheduled sleep episodes, ambient lighting was turned off. Illuminance was measured daily by a lux meter (Tektronix J17 Luma Color, Oregon, USA) in four locations positioned directly under light panels at 1.8 m from the floor. The room lighting was generated from ceiling-mounted 4100 K fluorescent lamps (Master TL5 HE 28 W/840 cool lights, Philips Lighting, Amsterdam, The Netherlands) covered with neutral density filters (three-stop LEE Filters, Lightmoves, Noble Park, Australia).

### Blood sample collection and processing

Blood was collected during the CR/CP wake intervals via an indwelling intravenous cannula, inserted into the forearm or antecubital vein approximately 1 hour after wake. Blood was collected every 2 hours, starting 2 hours after wake (Fig. 6). Whole blood was collected in a syringe and aliquoted into a blood tube spray coated with dipotassium ethylenediaminetetraacetic acid (K2EDTA). Samples were immediately centrifuged at 4°C or stored in a fridge at 4°C for no more than 30 min before processing. Once spun, plasma supernatant was aliquoted, snap-frozen in dry ice, and stored at −80°C for metabolomic analysis.

### Metabolomics analysis

For metabolomic extraction, plasma samples were thawed on ice in 20-μl aliquots and extracted using 180 μl of acetonitrile/methanol (1:1, v/v) solution containing 2 μM ^13^C-sorbitol, 2 μM ^13^C^15^N-AMP (adenosine monophosphate), and 2 μM ^13^C^15^N-UMP (uridine monophosphate) as internal standards. Samples were vortexed for 30 s, sonicated for 5 min at 4°C, and then incubated for 10 min at 4°C (in an Eppendorf Thermomixer). Samples were then centrifuged (4500*g*, 10 min at 4°C), and 100 μl of the supernatant was transferred into a glass vial. The pooled biological controls (PBQCs) contained a 10-μl aliquot of each sample extract. Sample extracts (7 μl) were resolved on a ZIC-pHILIC column (5-μm particle size, 150 mm by 4.6 mm, Merck SeQuant) connected to an Agilent 1290 (Santa Clara, CA, USA) high-performance liquid chromatography system running a 33-min gradient with mobile phases 20 mM ammonium carbonate (pH 9.0; Sigma-Aldrich; solvent A) and 100% acetonitrile (solvent B) at a constant flow rate of 300 μl/min. Metabolites were detected by electrospray ionization using an Agilent 6545 Q-TOF MS system (Santa Clara, CA, USA) in negative all-ion fragmentor mode, which included three collision energies (0, 10, and 20 V). For each experiment, LC-MS processing was conducted in one analytical batch, with samples randomized by participant and time, with a PBQC run every eighth sample to monitor instrument performance during the run. Solvent blanks were analyzed every 12 hours to monitor instrument performance. The instrument was cleaned and calibrated weekly to ensure a mass accuracy of ±0.2 parts per million (ppm). Detailed quadrupole–time-of-flight (Q-TOF) MS parameters are available (*60*).

### Data analysis

Data analysis for this study was designed to develop a biomarker that could be deployed in operational/point-of-care settings. The two sleep deprivation experiments used an identical laboratory protocol but were run as independent LC-MS experiments. These independent datasets were allocated to model training or testing, providing biologically and technically independent datasets for model hold-out analysis (experiment 1 training/experiment 2 testing for main analysis, experiment 2 training/experiment 1 testing for sanity check).

### LC-MS data cleaning, feature filtering and identification

#### LCMS data cleanup

Relative abundances based on area under the feature peak were obtained using MassHunter Quantitative Analysis B 0.8.00 for TOF (Agilent). An integrated matrix of 1035 true peaks was then generated by XCMS centWave algorithm (*61*) to detect features. Features with >20% zero values were removed, resulting in 929 features to be included in subsequent analyses. These data were imputed for left-censored missing value using QRLIC and were then median-normalized within sample. This is a standard approach to account for variation across the LC-MS run. These data were not further transformed for between-participant analyses. For within-participant analyses, a *z*-score for each feature was generated for each participant across time points to reduce between-participant variation.

#### Filtering of LCMS features

Data filtering and analyses were performed using R version 4.1.2 (https://r-project.org). Data were initially filtered by testing for fit to a general linear model (lm[stat]) and cosinor model with 24-hour period (cosinor.lm[cosinor]) in each individual and at the group level (lmFit[limma], LimoRhyde[limma]). To pass this filter and be included in modeling for biomarker candidates, features were required to have a significant and monotonic linear model in more than 50% of participants and at the group level (*P* < 0.05) in the training experiment (experiment 1 for main analysis, experiment 2 for sanity check) but not display a significant cosinor amplitude in more than 25% of participants (*P* < 0.05). Filtering in this way was important as significant responses at the group level are not always reflected in individual trends (*26*) such that biomarker development on erroneous group-level trends can be misleading. Features displaying a 24-hour cycle in more than a quarter of participants were also excluded as they are likely under circadian control. For future implementation of a biomarker, it is difficult to control for an individual’s circadian timing as samples may be collected across multiple circadian phases, even if collected at the same clock time (*62*). Hence, a sleep deprivation biomarker should ideally be under control of the sleep homeostat (linear change) and not under circadian control (cyclical).

Filtered feature peaks were confirmed in MassHunter using the “compounds at a glance” function. The MassHunter metabolite area data for filtered features were also visualized to ensure any batch effects, XCMS processing, and normalization of the data had not introduced unwanted variation in the filtered features.

#### Identification of filtered features

Identification was attempted for all features that passed data filtering using accurate mass, RT, and MS/MS fragmentation patterns for each feature. The identification and evaluation of metabolomics data from LC-MS (IDEOM) workflow was used to calculate database RTs based on included standards from the experiments to support mass-based identification (*63*). In addition to the IDEOM workflow, online databases were accessed to search masses to assign formulae and identify features (https://lipidmaps.org/; http://hmdb.ca/; https://biocyc.org/; http://chemspider.com/; https://mzcloud.org/). Standards were run where available to discriminate between compounds and isomers to achieve level 1 identification as per the Metabolomics Standards Initiative (*64*). Where isomers could not be discriminated or standards could not be run, fragmentation patterns, *m*/*z*, and RT matches resolved putative identification to a higher level of classification.

### Modeling of biomarker candidates

Random forest models are nonlinear multivariate techniques based on decision trees, which estimate the relative importance of each predictor variable (LC-MS features) on the response variable (TSW or WR-SD). This machine learning approach was selected because it is useful for biomarker discovery, as it is nonparametric and allows for nonlinear relationships between variables in complex biological data [e.g., (*65*–*68*)].

Random forest models were trained on experiment 1 data (12 participants) and tested on experiment 2 (11 participants) in a hold-out analysis (randomForest[randomForest]) (*69*). Seed was set at 123 for all analyses, with model parameters mtry = 3, ntree = 500 for all models; minimum size of terminal nodes was 1 for classification models and 5 for regression models. Sanity check models were also built (trained on experiment 2 data and tested on experiment 1). Candidates were selected in the training experiment from the filtered features using the VSURF algorithm (parameters: ntree.thres = 300, nfor.thres = 40, ntree.interp = 100, nfor.interp = 30, ntree.pred = 100, nfor.pred = 10), which progressively removed variables and estimated the reduction in cross-validated performance of random forest models (*70*). The final biomarker candidates were VSURF variables that were selected across most models [model type (classification/regression) and data type (within/between participant)].

For predicting WR-SD, a training set of 182 samples from 12 participants across two classes (93 samples WR and 89 samples SD) was used to build a classification random forest models, which were evaluated using the model accuracy and confusion matrices. Variables were ranked by relative variable importance based on mean decrease accuracy (MDA) on variable removal. Model accuracy with lower-upper 95% CI was assessed as 1 − out of bag error for training models and prediction accuracy for testing models. Confusion matrices from the final model were used to define PPVs and NPVs. Predicted values from the classification random forests were then used to define ROC curves to visualize the predictive accuracy as SP and SN and to calculate the AUC (with lower and upper 95% CI) of the models (roc[pROC]). To predict TSW, a training set of 218 samples from 12 participants across 19 time points (10 to 12 samples per time point) was used to build a regression random forest model. Model performance was evaluated using R^2^, and RMSE to interpret variance and relative variable importance was estimated by the percent increase in mean squared error (MSE%) on variable removal.

Final biomarker models were then tested on experiment 2. For predicting WR-SD, a test set of 85 WR samples and 84 SD samples was assessed. Furthermore, the same training models were tested on each participant in the test data to assess individual-level prediction (TSW: 19 samples per participant, except participants U: 14, V: 17, and W: 16 samples; WR-SD: 8 samples per participant per group, except participants U and V: 7 per group and W: 7 WR and 6 SD). To predict TSW, this provided 198 samples from 11 participants across 19 time points (9 to 11 samples per time point).

The accuracy of models to predict sleep deprivation (>24 hours TSW) and extended wake (increasing TSW) using fewer variables were assessed by building classification and regression models with all possible combinations of two or more of the five consistently selected features (26 combinations). These analyses allowed for a more detailed examination of model resilience and flexibility. Multiple comparisons (Bonferroni adjustment) were used to assess the significance of these models.

To investigate changes in model accuracy when assessing classifications including samples from outside the clock time–matched intervals (e.g., 18 to 22 hours), classification models were also built closing the distinction between WR (0 to 16 hours) and SD (24 to 38 hours) conditions, by including 18-, 20-, and 22-hour measurements in each condition (WR or SD) in a stepwise manner, culminating in the closest distinction of WR (<24 hours) compared with SD (>24 hours). Furthermore, to investigate a lower level of sleep loss, classification models were also built comparing WR condition with only samples from within the biological night [e.g., WR (0 to 16 hours TSW) compared with SD (18 to 24 hours TSW)].

Last, the inclusion of the matched control in this study helped to separate the impact of increasing time awake from inactivity, light, meal timings, and environment, which may also change systematically/incrementally across the CR protocol. As the matched control had comparable inactivity/environmental settings, the opposite/lack of change observed (during sleep deprivation) in biomarker candidates following a sleep interval would provide further evidence that the biomarker is indicative of sleep deprivation. To assess this, the matched control samples (5 participants, 15 samples per condition) were compared with time-matched sleep deprivation samples from experiment 1 (12 participants, 33 to 36 samples per condition) and experiment 2 (11 participants, 21 to 22 samples per condition). These data were assessed in either a clock time-matched (2 to 6 hours TSW day 2 versus 2 to 6 hours day 3 for matched control and 2 to 6 hours versus 26 to 30 hours for sleep deprivation) or evening/morning design (12 to 16 hours TSW day 2 versus 2 to 6 hours day 3 for matched control and 12 to 16 hours versus 26 to 30 hours for sleep deprivation) using linear mixed model with participant as a random factor (lmer[lme4]) (*71*). Furthermore, the influence of meal timing on each biomarker candidate’s levels was assessed comparing WR samples from the matched control (CP, with three large meals and three snacks) and sleep deprivation (CR, with hourly snacks). Mixed linear models including TSW and study protocol (meal timing) as fixed factors and participant as a random factor were used to compare trends in *z*-scored candidate data across a WR day (day 3 of matched control versus WR CR from sleep deprivation).

## Supplementary Materials

### This PDF file includes:

Figs. S1 to S8 Tables S1 to S7 References
