# Supplementary material for: Accurate detection of acute sleep deprivation using a metabolomic biomarker—A machine learning approach
Source: Sci Adv. 2024 Mar 8;10(10):eadj6834. doi: 10.1126/sciadv.adj6834 (PMC11094653; doi:10.1126/sciadv.adj6834)
Supplement: Supplementary file 1 — Figs. S1 to S8 Tables S1 to S7 References [file sciadv.adj6834_sm.pdf]

Science Advances
AAAS

Supplementary Materials for

Accurate detection of acute sleep deprivation using a metabolomic biomarker—A machine learning approach

Katherine Jeppe *et al.*

Corresponding author: Clare Anderson, clare.anderson@monash.edu

Sci. Adv. **10**, eadj6834 (2024)
DOI: 10.1126/sciadv.adj6834

This PDF file includes:

Figs. S1 to S8
Tables S1 to S7
References

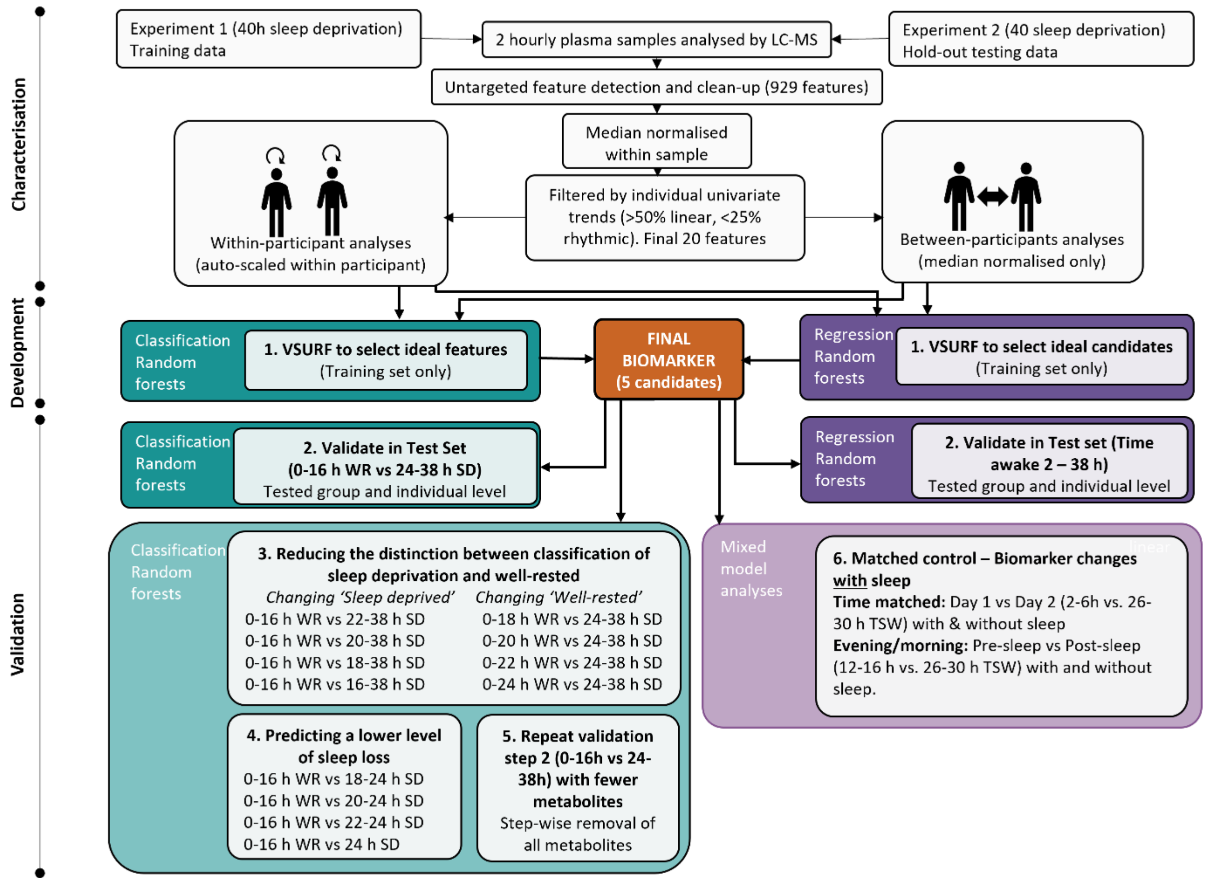

Figure S1: Graphical Representation of our Approach and Analysis Plan. Biomarkers were characterised by linear/circadian trends with time awake to reduce the final number of metabolites for development and modelling (only those with strong linear changes without circadian confounds). **[1]** For biomarker development, machine learning techniques identified the best candidates (using VSURF) to predict a level of sleep loss (classification) or time awake (regression). The 'best' five metabolites formed our final biomarker. **[2]** We validated our biomarker in an independent test set for both classification and regression, within and between subjects. **[3]** We assessed biomarker accuracy when classifying lower cut-offs of sleep deprivation (e.g., 22h, 20h etc) and less conservative cut-offs for well-rested (e.g., 18h, 20h). **[4]** We then examined accuracy with a lower, more real-world level of sleep loss (<16h versus 18-24h, i.e., day versus night only). **[5]** As some metabolites may not be useful in future, we re-tested the primary analyses [1] but with all possible metabolite combinations. **[6]** Finally, we checked the 'recovery' of the biomarker with a period of sleep using both circadian time matched AND pre/post sleep.

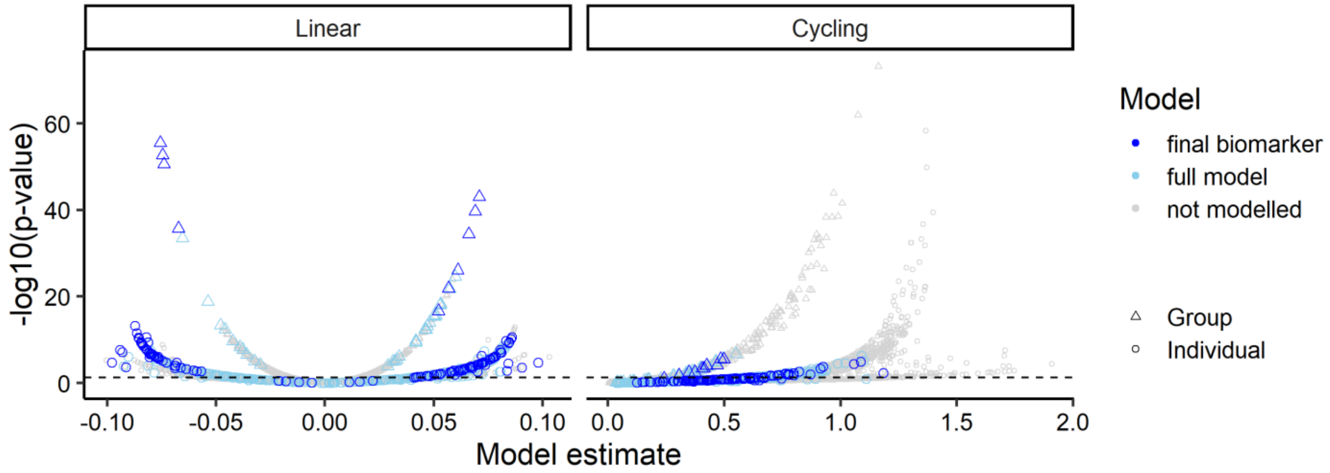

Figure S2. Volcano plots showing group and individual level significance (-$\log_{10}$(p-value)) against model estimate (slope for linear models and amplitude for cosinor models) of 929 features detected with hydrophilic interaction liquid chromatography-mass spectrometry (HILIC LC-MS). Symbol colours indicate if features were included in the final biomarker (dark blue), passed the data reduction filter (full model; light blue) or were not modelled (grey). Group models are displayed as triangles and individual models are displayed as circles. Significance at the p = 0.05 is indicated with a dashed line.

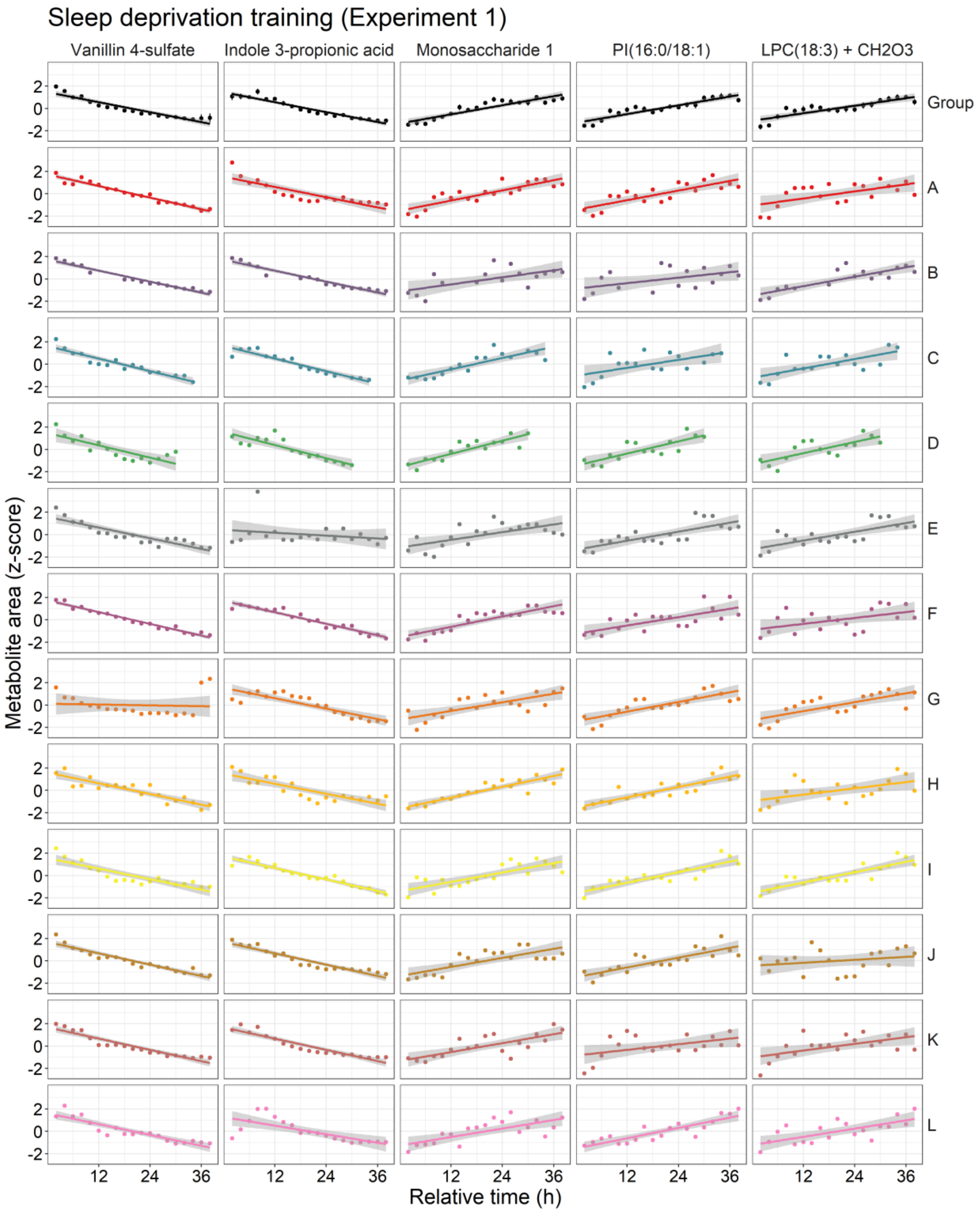

Figure S3. **Within-participant group and individual level trends of five consistently important metabolites across time since wake in Experiment 1 (sleep deprivation training).** Displaying metabolite peak area (median normalised z-scored within participant) and linear fit with shaded 95% CI for each participant (A-L) and **standard error bars for group points**.

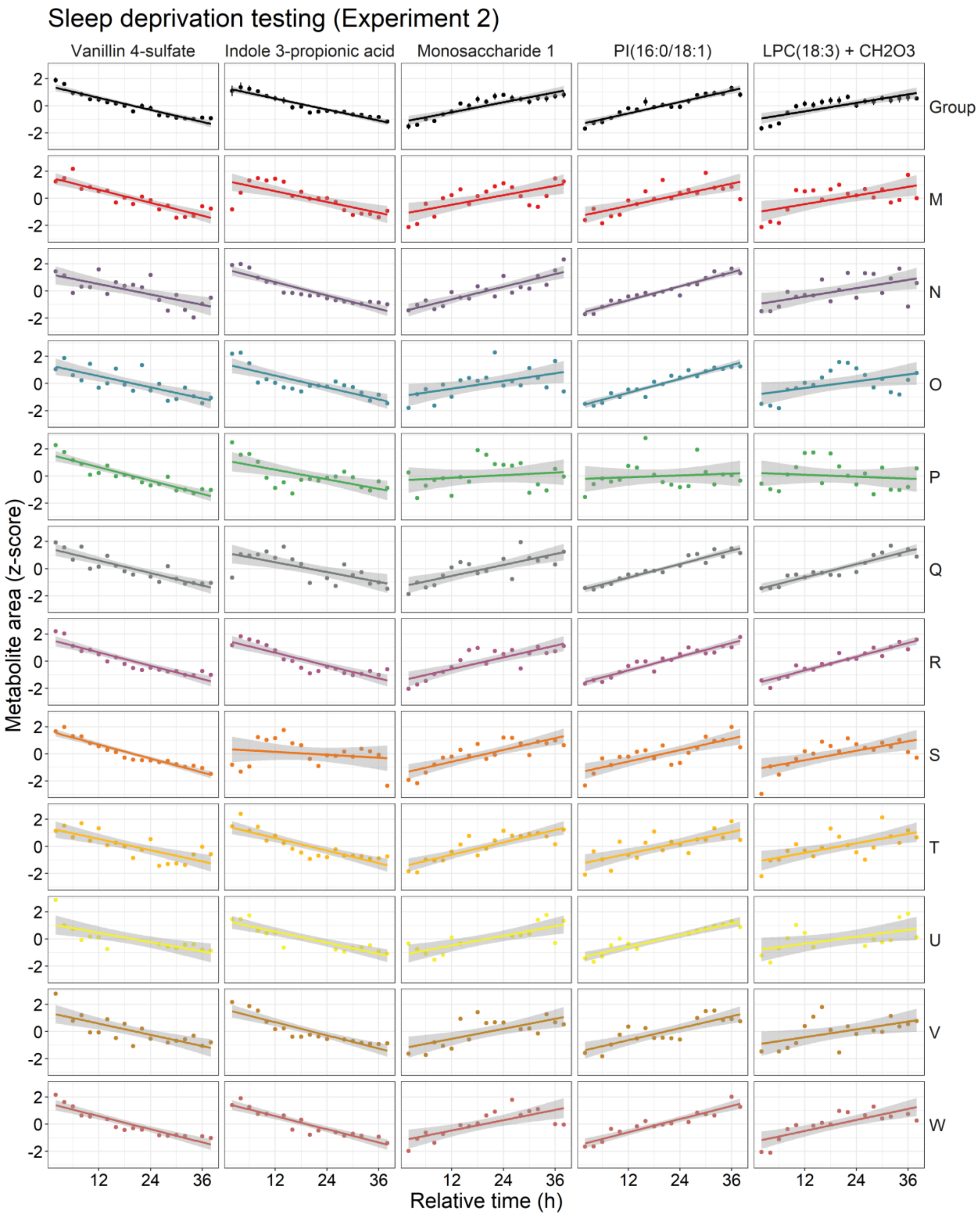

Figure S4. Within-participant group and individual level trends of five consistently important metabolites across time since wake in Experiment 2 (sleep deprivation testing). Displaying metabolite peak area (median normalised z-scored within participant) and linear fit with shaded 95% CI for each participant (M-W) and standard error bars for group points.

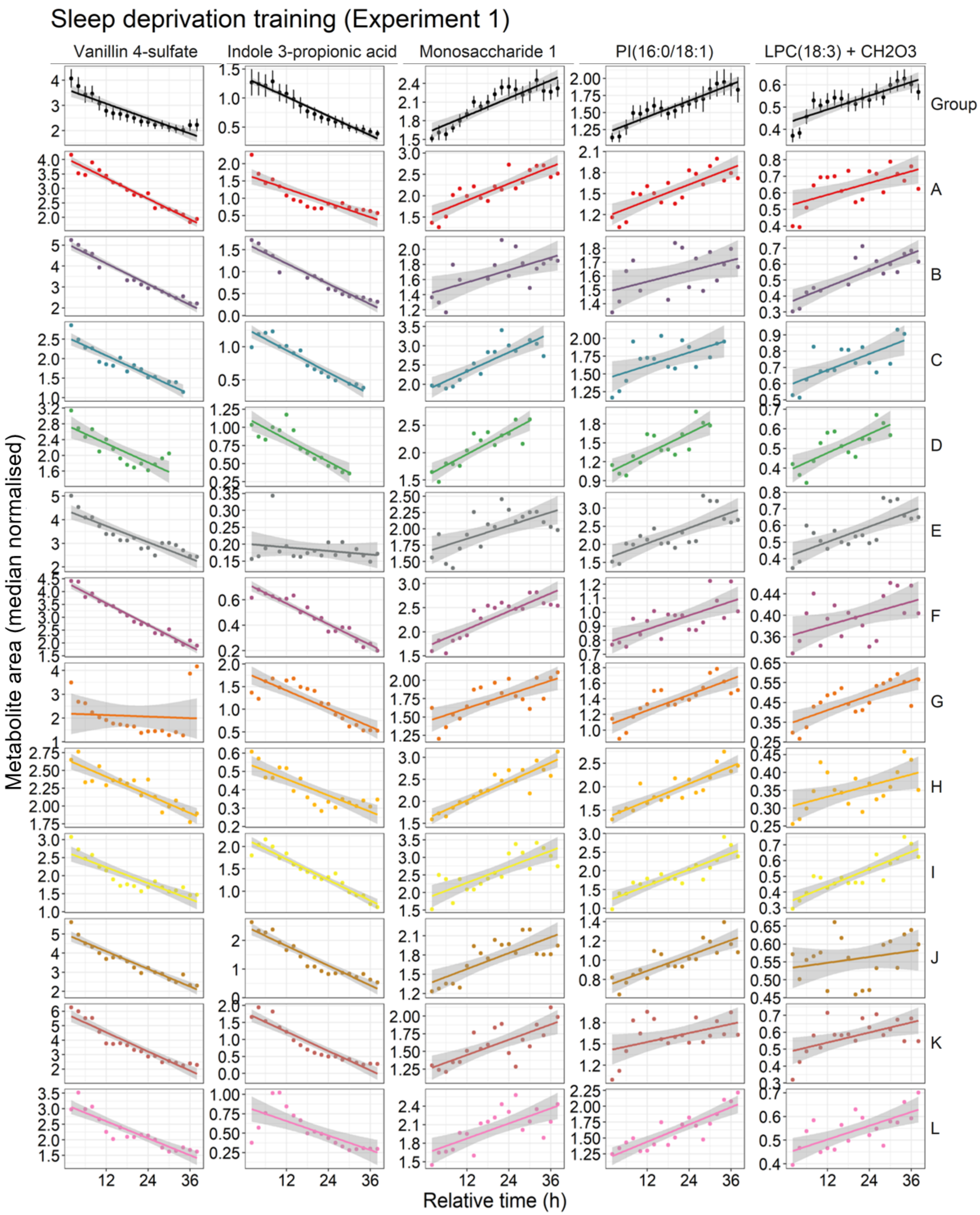

Figure S5. Between-participants group and individual level trends of five consistently important metabolites across time since wake in Experiment 1 (sleep deprivation training). Displaying metabolite peak area (median normalised) and linear fit with shaded 95% CI for each participant (A-L) and standard error bars for group points.

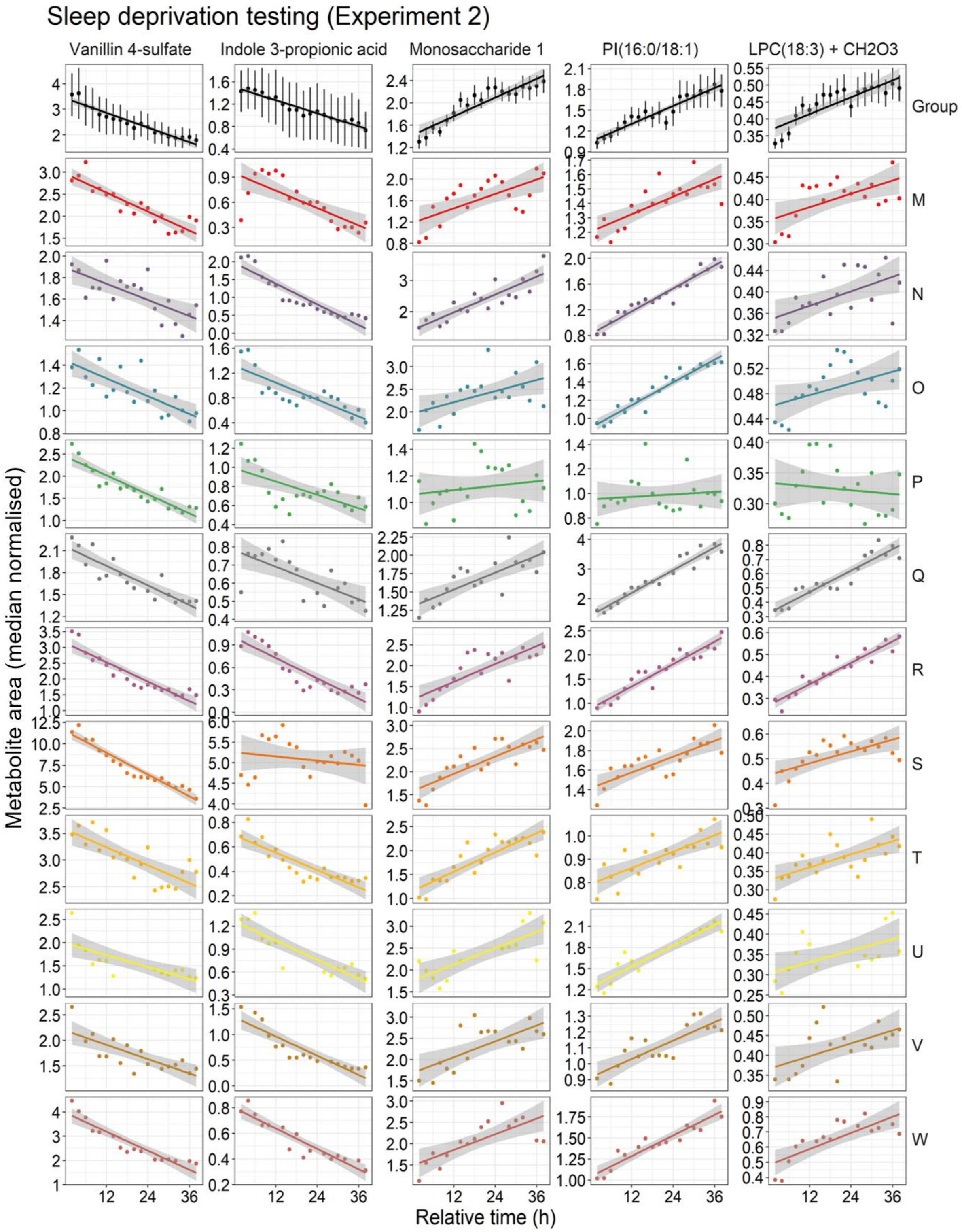

Figure S6. Between-participants group and individual level trends of five consistently important metabolites across time since wake in Experiment 2 (sleep deprivation testing). Displaying metabolite peak area (median normalised) and linear fit with shaded 95% CI for each participant (M-W) and standard error bars for group points.

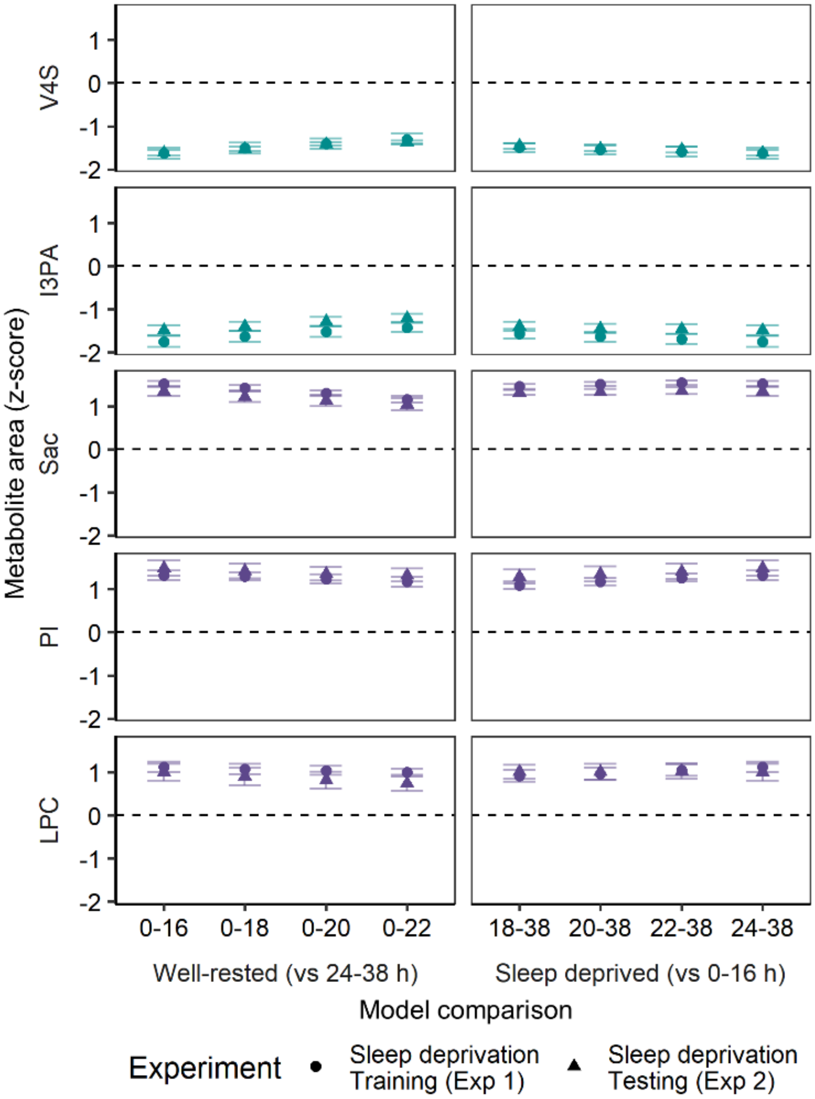

Figure S7. Magnitude of change (expressed as a z-score) in metabolite features as a function of sleep deprivation. *Left Panels:* Relative to sleep deprivation (24-38h), group level changes for varying levels of well-rested ranging from typical well-rested state (0-16h) to less conservative well-rested classifications. *Right Panels*: Relative to well-rested (0-16h), group level changes for varying levels of sleep deprivation thresholds ranging from 18hours and beyond to 24hours and beyond.

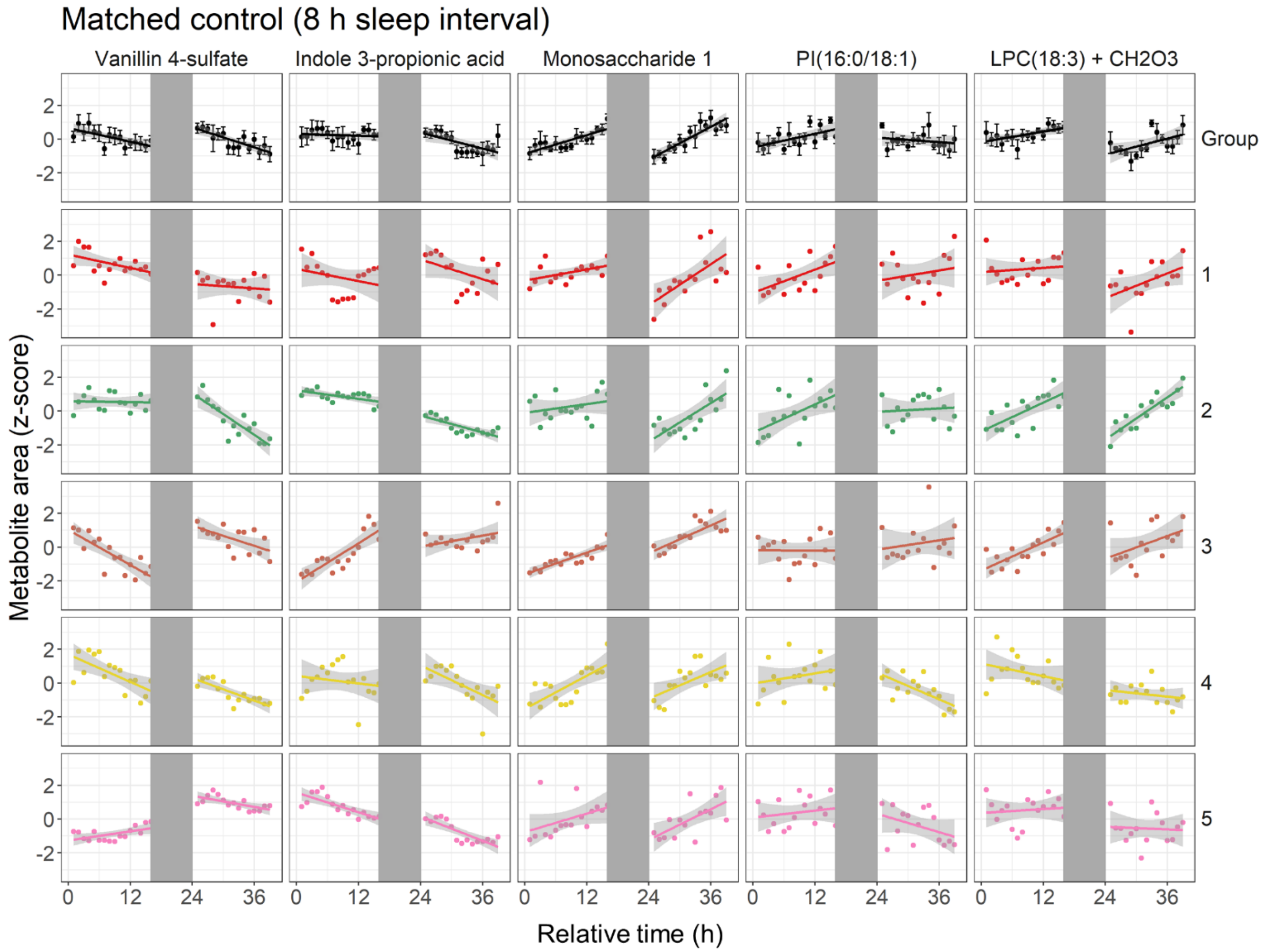

Figure S8. Within-participant group and individual level trends of five consistently important metabolites across time since wake in Matched Control experiment. Displaying metabolite peak area (median normalised z-scored within participant) and linear fit with shaded 95% CI for each participant (1-5) and standard error bars for group points. Grey band from 16 - 24 relative Time (h) indicates an 8 h sleep opportunity.

Table S1.

Filtered HILIC LC-MS features included in predictive modelling. Including mass to charge ratio (m/z) and retention time (RT), calculated formulas, ppm difference between formula and candidate m/z, putative identified name, and level of identification reached as described by the metabolite standards initiative, features in bold were confirmed to level 1 identification using chemical standards.

Putative identified name	Feature name	m/z	Accurate mass	RT (min)	Formula	ppm	Level
**Vanillin 4-sulfate**	**Unidentified 262**	**230.9955**	**232.0028**	**4.0**	**C8H8SO6**	**-5.9**	**1**
**Indole 3-propionate**	**Unidentified_181**	**188.0722**	**189.0795**	**7.3**	**C11H11NO2**	**2.5**	**1**
Monosaccharide 1	Unidentified 158	179.0561	180.0633	13.3	C6H12O6	-0.3	3
**PI(16:0/18:1)**	**Unidentified_906**	**835.5352**	**836.5425**	**3.3**	**C43H81O13P**	**1.2**	**1**
(7S,8S)-DiHODE	Unidentified 394	311.2221	312.2293	4.0	C18H32O4	-2.3	3
**4-Pyridoxate**	**Unidentified_165**	**182.0458**	**183.0531**	**4.5**	**C8H9NO4**	**-0.2**	**1**
pABA-Glc	Unidentified 368	298.0966	299.1039	13.7	C13H17NO7	11.4	3
**LPC(18:3) + CH2O3**	**Unidentified_753**	**578.3101**	**579.3174**	**4.1**	**C27H50NO10P**	**0.3**	**1**
Indan-1-ol	Unidentified_86	133.0657	134.0729	3.9	C9H10O	-1.4	2
Mercaptopropanoic acid +FA	Unidentified 112	151.0072	152.0144	12.2	C4H8O4S	-7.9	2
Monosaccharide analogue 1	Unidentified_135	165.0769	166.0841	12.7	C6H14O5	0.2	3
PS(O-20:0/22:6)	Unidentified_919	848.5975	849.6048	3.5	C48H84NO9P	19.3	3
2-Keto-glutamarate	Unidentified_99	144.0303	145.0375	12.8	C5H7NO4	0.1	2
PI(36:1)	Unidentified_934	863.5627	864.5699	3.3	C45H85O13P	3.6	3
DGCC(20:5/20:5)	Unidentified_935	864.5655	865.5728	3.3	C50H77NO8	3.8	2
**L-Proline**	**X1.Proline**	**114.0562**	**115.0635**	**14.6**	**C5H9NO2**	**0**	**1**
L-Proline C13	Unidentified_58	115.0591	116.0664	14.6	C5H9NO2(13C)	0	2
PE(40:5)	Unidentified_879	792.5442	793.5514	3.5	C45H80NO8P	2.6	3
PE(40:5) C13	Unidentified 880	793.5487	794.5560	3.5	C45H80NO8P(13C)	1.9	3
Monosaccharide 2	Unidentified_222	209.0664	210.0736	13.9	C7H14O7	-1.4	3

Table S2.

Results from regression (predicting time since wake) and classification (predicting clock time matched >24 h awake (0-16 h to 24-38 h awake)) random forest models for within-participant and between-participant analyses. Showing models trained on Experiment 1 (tested Experiment 2) and sanity checks (trained on Experiment 2 and tested on Experiment 1). Displaying the number of features used to build the models (all filtered features (20 or 13[a]), variables selected with the VSURF variable selection tool (various[v]), and the five final biomarker candidates (final[f])). For regression models, variance explained (as R^2) and root mean square error (RMSE) are displayed. For classification models, model accuracy (Lower – Upper 95% CI), Area Under the receiver operating Curve (AUC, Lower – Upper 95% CI), negative and positive prediction accuracy (NPV and PPV%), specificity (SP%) and sensitivity (SN%) are displayed.

Training Experiment	Comparison	Model	Variables (Classification)	Tree depth (Regression)	R^2 (%)	RMSE	Variables (Classification)	Tree depth (Classification)	Accuracy (%) (Lower - Upper)	AUC (%) (Lower - Upper)	SP (%)	SN (%)	NPV (%)	PPV (%)
Experiment 1	Within-participant	Training[a]	20	171	98.6	1.27	20	35	98.4 (95.3 - 99.7)	99.9 (99.7 - 100)	100	96.7	96.8	100
		Testing[a]	20		86.1	4.14	20		95.9 (91.7 - 98.3)	99.4 (98.7 - 100)	96.4	95.3	95.3	96.4
		Training[v]	9	173	98.4	1.35	6	35	97.8 (94.5 - 99.4)	99.8 (99.4 - 100)	98.9	96.7	96.8	98.9
		Testing[v]	9		87.2	3.98	6		96.4 (92.4 - 98.7)	99.4 (98.7 - 100)	97.6	95.3	95.3	97.6
		Training[f]	5	159	98.0	1.52	5	35	97.8 (94.5 - 99.4)	99.7 (99.3 - 100)	98.9	96.7	96.8	98.9
		Testing[f]	5		86.7	4.06	5		94.7 (90.1 - 97.5)	99.2 (98.3 - 100)	96.3	93.1	92.9	96.4
	Between-participants	Training[a]	20	167	96.5	2.03	20	53	95.1 (90.8 - 97.7)	98.6 (97.1 - 100)	96.7	93.5	93.5	96.6
		Testing[a]	20		32.6	9.12	20		72.8 (65.4 - 79.3)	88.4 (83.5 - 93.4)	67.3	83.9	89.4	56.0
		Training[v]	13	165	96.6	2.01	14	53	95.1 (90.8 - 97.7)	98.9 (97.6 - 100)	95.7	94.4	94.6	95.5
		Testing[v]	13		39.3	8.66	14		72.2 (64.8 - 78.8)	90.1 (85.6 - 94.6)	67.0	82.5	88.2	56.0
		Training[f]	5	171	94.0	2.66	5	53	92.9 (88.1 - 96.1)	97.7 (95.8 - 99.6)	94.4	91.3	91.4	94.4
		Testing[f]	5		48.2	8.00	5		79.3 (72.4 - 85.1)	89.1 (84.4 - 93.8)	76.0	83.6	85.9	72.6
Experiment 2	Within-participant	Training[a]	20	157	97.9	1.63	20	33	95.9 (91.7 - 98.3)	99.5 (99 - 100)	96.4	95.3	95.3	96.4
		Testing[a]	20		86.4	4.00	20		96.7 (93 - 98.8)	99.7 (99.3 - 100)	96.8	96.6	96.8	96.6
		Training[v]	14	155	97.8	1.63	6	31	96.4 (92.4 - 98.7)	99 (97.8 - 100)	97.6	95.3	95.3	97.6
		Testing[v]	14		86.8	3.95	6		95.1 (90.8 - 97.7)	99.6 (99.1 - 100)	96.7	93.5	93.5	96.6
		Training[f]	5	145	97.2	1.86	5	31	96.4 (92.4 - 98.7)	99.5 (98.8 - 100)	97.6	95.3	95.3	97.6
		Testing[f]	5		89.6	3.49	5		97.8 (94.5 - 99.4)	99.7 (99.3 - 100)	100	95.7	95.7	100
	Between-participants	Training[a]	13	147	95.8	2.28	13	55	91.7 (86.5 - 95.4)	97.8 (96 - 99.6)	91.8	91.7	91.8	91.7
		Testing[a]	13		46.3	7.95	13		84.6 (78.5 - 89.5)	89.5 (84.4 - 94.7)	83.5	85.9	87.1	82.0
		Training[v]	7	147	95.8	2.29	8	55	91.7 (86.5 - 95.4)	97.9 (96.2 - 99.6)	92.8	90.7	90.6	92.9
		Testing[v]	7		42.5	8.23	8		79.1 (72.5 - 84.8)	88.2 (82.9 - 93.5)	75.7	84.0	87.1	70.8
		Training[f]	5	145	94.4	2.62	5	51	91.1 (85.8 - 94.9)	96.9 (94.6 - 99.2)	93.8	88.8	88.2	94.0
		Testing[f]	5		52.7	7.46	5		80.8 (74.3 - 86.2)	90.7 (86.6 - 94.9)	81.5	80.0	80.6	80.9

Table S3.

Results from sanity check analysis (trained on Experiment 2 and tested on Experiment 1) for group and individual level regression (predicting time since wake) and classification (predicting clock matched >24 h awake (0-16 h to 24-38 h TSW)) random forest models for within-participant and between-participant analyses built with the five final biomarker candidates. For regression models, variance explained (as R^2) and root mean square error (RMSE) are displayed (Tree depth was 145, for both within-participant and between participants regression training models). For classification models, model accuracy (Lower – Upper 95% CI), Area Under the receiver operating Curve (AUC, Lower – Upper 95% CI), negative and positive prediction accuracy (NPV and PPV%), specificity (SP%) and sensitivity (SN%) are displayed (Tree depth was 31 and 51, for within-participant and between participants classification training models, respectively). Note if PPV or NPV are 0 SN and SP cannot be calculated.

Comparison	Model	Participant	Accuracy (%) (Lower - Upper)	AUC (%) (Lower - Upper)	SP (%)	SN (%)	NPV (%)	PPV (%)	R^2 (%)	RMSE
Within-participant	Training	Group	96.4 (92.4 - 98.7)	99.5 (98.8 - 100)	97.6	95.3	95.3	97.6	97.2	1.86
	Testing	Group	97.8 (94.5 - 99.4)	99.7 (99.3 - 100)	100	95.7	95.7	100	89.6	3.49
		A	100 (79.4 - 100)	100 (100 - 100)	100	100	100	100	93.7	2.74
		B	100 (75.3 - 100)	100 (100 - 100)	100	100	100	100	86.1	4.31
		C	100 (75.3 - 100)	100 (100 - 100)	100	100	100	100	86.1	3.65
		D	91.7 (61.5 - 99.8)	100 (100 - 100)	100	80.0	87.5	100	66.6	4.99
		E	100 (79.4 - 100)	100 (100 - 100)	100	100	100	100	86.8	3.98
		F	100 (79.4 - 100)	100 (100 - 100)	100	100	100	100	94.3	2.61
		G	87.5 (61.7 - 98.4)	96.9 (89.6 - 100)	100	80.0	75.0	100	78.4	5.09
		H	100 (79.4 - 100)	100 (100 - 100)	100	100	100	100	95.5	2.33
		I	100 (79.4 - 100)	100 (100 - 100)	100	100	100	100	96.3	2.11
		J	100 (79.4 - 100)	100 (100 - 100)	100	100	100	100	92.3	3.05
		K	93.8 (69.8 - 99.8)	98.4 (94.1 - 100)	100	88.9	87.5	100	89.1	3.62
		L	100 (79.4 - 100)	100 (100 - 100)	100	100	100	100	96.3	2.11
Between-participants	Training	Group	91.1 (85.8 - 94.9)	96.9 (94.6 - 99.2)	93.8	88.8	88.2	94.0	94.4	2.62
	Testing	Group	80.8 (74.3 - 86.2)	90.7 (86.6 - 94.9)	81.5	80.0	80.6	80.9	52.7	7.46
		A	100 (79.4 - 100)	100 (100 - 100)	100	100	100	100	68.5	6.15
		B	69.2 (38.6 - 90.9)	100 (100 - 100)	55.6	100	100	50.0	51.6	8.05
		C	76.9 (46.2 - 95)	100 (100 - 100)	100	62.5	62.5	100	31.6	8.08
		D	91.7 (61.5 - 99.8)	100 (100 - 100)	100	80.0	87.5	100	81.3	3.74
		E	50 (24.7 - 75.3)	92.2 (76.2 - 100)			0.0	100	4.4	10.71
		F	100 (79.4 - 100)	100 (100 - 100)	100	100	100	100	83.0	4.51
		G	68.8 (41.3 - 89)	98.4 (94.1 - 100)	61.5	100	100	37.5	52.5	7.55
		H	87.5 (61.7 - 98.4)	100 (100 - 100)	100	80.0	75.0	100	57.6	7.13
		I	93.8 (69.8 - 99.8)	100 (100 - 100)	100	88.9	87.5	100	70.9	5.91
		J	50 (24.7 - 75.3)	43.8 (12.3 - 75.2)	50.0		100	0.0	-14.5	11.72
		K	87.5 (61.7 - 98.4)	100 (100 - 100)	80.0	100	100	75.0	69.3	6.07
		L	93.8 (69.8 - 99.8)	100 (100 - 100)	100	88.9	87.5	100	69.4	6.06

Table S4.

Testing model results from classification random forests for within and between-participant analyses. Showing models trained on Experiment 1 and tested Experiment 2 and sanity checks (trained on Experiment 2 and tested on Experiment 1). Classification models predicting clock matched >24 h awake were built and tested closing the distinction between well-rested and sleep deprived classification by altering the Time grouping (h). Models were built with the five consistent features across VSURF selected models. Model accuracy (Lower – Upper 95% CI), Area Under the receiver operating Curve (AUC, Lower – Upper 95% CI), negative and positive prediction accuracy (NPV and PPV%), specificity (SP%) and sensitivity (SN%) are displayed. For the training models tested here, tree depth ranged from 27-73 nodes).

Training Experiment	Comparison	Time grouping (h)	Accuracy (%) (Lower - Upper)	AUC (%) (Lower - Upper)	SP (%)	SN (%)	NPV (%)	PPV (%)
Experiment 1	Within-participant	0-16 vs 18-38	92.4 (87.8 - 95.7)	98 (96.6 - 99.4)	91.7	93.0	90.6	93.8
		0-16 vs 20-38	93.6 (89.1 - 96.7)	98.9 (98.1 - 99.8)	92.9	94.2	92.9	94.2
		0-16 vs 22-38	93.8 (89.2 - 96.9)	98.8 (97.9 - 99.8)	93.0	94.6	94.1	93.5
		0-18 vs 24-38	96.6 (92.8 - 98.8)	99.1 (98.1 - 100)	98.9	94.3	94.7	98.8
		0-20 vs 24-38	92.1 (87.2 - 95.5)	97.9 (96.3 - 99.5)	95.0	88.8	90.5	94.0
		0-23 vs 24-38	90.4 (85.4 - 94.1)	96.5 (94.2 - 98.7)	90.6	90.1	93.0	86.9
	Between-participants	0-16 vs 18-38	70.2 (63.3 - 76.5)	84.7 (79.4 - 90)	64.8	74.5	67.1	72.6
		0-16 vs 20-38	72.3 (65.4 - 78.6)	85.8 (80.6 - 91)	67.7	76.8	74.1	70.9
		0-16 vs 22-38	75.3 (68.3 - 81.4)	87.6 (82.7 - 92.5)	71.1	80.2	81.2	69.9
		0-18 vs 24-38	78.2 (71.4 - 84)	88.4 (83.7 - 93.1)	76.9	80.0	84.2	71.4
		0-20 vs 24-38	77.2 (70.6 - 83)	88.1 (83.5 - 92.7)	78.7	75.3	81.0	72.6
		0-23 vs 24-38	76.8 (70.3 - 82.5)	87.4 (82.8 - 92.1)	79.3	73.2	80.7	71.4
Experiment 2	Within-participant	0-16 vs 18-38	93.6 (89.5 - 96.4)	98.7 (97.6 - 99.8)	92.5	94.4	92.5	94.4
		0-16 vs 20-38	96.1 (92.5 - 98.3)	99.5 (98.8 - 100)	96.7	95.7	94.6	97.3
		0-16 vs 22-38	96.9 (93.4 - 98.9)	99.7 (99.2 - 100)	98.9	95.2	94.6	99.0
		0-18 vs 24-38	95.9 (92 - 98.2)	99.3 (98.6 - 100)	98.0	93.5	94.3	97.8
		0-20 vs 24-38	93.7 (89.5 - 96.6)	98.9 (98 - 99.8)	95.6	91.3	93.2	94.4
		0-23 vs 24-38	90.4 (85.7 - 93.9)	96.6 (94.7 - 98.6)	92.9	87.0	90.7	89.9
	Between-participants	0-16 vs 18-38	76.1 (69.9 - 81.6)	86.7 (82.2 - 91.3)	72.0	79.2	72.0	79.2
		0-16 vs 20-38	77.2 (70.8 - 82.7)	88.3 (84 - 92.7)	75.0	78.9	74.2	79.6
		0-16 vs 22-38	77.8 (71.3 - 83.5)	89.6 (85.4 - 93.8)	77.2	78.4	76.3	79.2
		0-18 vs 24-38	78.4 (71.9 - 83.9)	89 (84.4 - 93.6)	79.4	77.0	81.0	75.3
		0-20 vs 24-38	77.7 (71.4 - 83.2)	86 (81 - 91.1)	78.9	75.9	82.9	70.8
		0-23 vs 24-38	77.5 (71.4 - 82.9)	84.6 (79.3 - 89.9)	80.3	73.3	82.2	70.8

Table S5.

Testing model results from classification random forest models for within- and between-participants analyses. Showing models trained on Experiment 1 and tested Experiment 2 and sanity checks (trained on Experiment 2 and tested on Experiment 1). Classification models predicting well-rested (0-16 h TSW) vs stepwise biological night classifications (time grouping) for the sleep deprived group. Models were built with the five consistent variables across VSURF selected models). Model accuracy (Lower – Upper 95% CI), Area Under the receiver operating Curve (AUC, Lower – Upper 95% CI), negative and positive prediction accuracy (NPV and PPV%), specificity (SP%) and sensitivity (SN%) are displayed. For the training models tested here, tree depth ranged from 19 to 57 nodes).

Training Experiment	Comparison	Time grouping (h)	Accuracy (%) (Lower - Upper)	AUC (%) (Lower - Upper)	SP (%)	SN (%)	NPV (%)	PPV (%)
Experiment 1	Within-participant	0-16 vs 18-24	84.6 (76.9 - 90.4)	95.1 (91.8 - 98.4)	89.3	74.4	88.2	76.3
		0-16 vs 20-24	91.2 (84.3 - 95.7)	97.5 (95.3 - 99.8)	94.1	82.1	94.1	82.1
		0-16 vs 22-24	93.2 (86.5 - 97.2)	97.6 (95.2 - 100)	95.3	82.4	96.5	77.8
		0-16 vs 24	96.8 (91 - 99.3)	98.7 (96.8 - 100)	97.7	87.5	98.8	77.8
	Between-participants	0-16 vs 18-24	69.1 (60.1 - 77.1)	76.9 (68.2 - 85.5)	81.3	50.0	71.8	63.2
		0-16 vs 20-24	73.5 (64.3 - 81.3)	79.1 (70.1 - 88.1)	84.8	47.1	78.8	57.1
		0-16 vs 22-24	78.6 (69.5 - 86.1)	80.2 (69.6 - 90.7)	89.9	41.7	83.5	55.6
		0-16 vs 24	80.9 (71.4 - 88.2)	83.5 (72.2 - 94.7)	94.7	26.3	83.5	55.6
Experiment 2	Within-participant	0-16 vs 18-24	89.4 (83.1 - 93.9)	96.9 (94.4 - 99.4)	89.0	90.2	95.7	77.1
		0-16 vs 20-24	93 (87.2 - 96.8)	98.5 (96.9 - 100)	92.9	93.5	97.8	80.6
		0-16 vs 22-24	93.2 (87 - 97)	98.5 (96.9 - 100)	93.8	90.0	97.8	75.0
		0-16 vs 24	94.3 (88 - 97.9)	99.3 (98.1 - 100)	94.8	87.5	98.9	58.3
	Between-participants	0-16 vs 18-24	74.5 (66.4 - 81.4)	78.4 (70.2 - 86.7)	80.6	62.5	80.6	62.5
		0-16 vs 20-24	79.8 (71.9 - 86.4)	80.7 (71.8 - 89.7)	84.5	65.6	88.2	58.3
		0-16 vs 22-24	86.3 (78.7 - 92)	82.2 (72.3 - 92.1)	90.5	68.2	92.5	62.5
		0-16 vs 24	93.3 (86.7 - 97.3)	81.7 (65.2 - 98.3)	94.8	77.8	97.8	58.3

Table S6.

Results from regression (predicting time since wake) and classification (predicting clock matched >24 h awake (0-16 h to 24-38 h awake)) random forest models for within-participant and between-participant analyses using subset variables from the final biomarker. Showing models trained on Experiment 1 (tested Experiment 2). Displaying the number of features used to build the models. For classification models, model accuracy (Lower – Upper 95% CI), Area Under the receiver operating Curve (AUC, Lower – Upper 95% CI), negative and positive prediction accuracy (NPV and PPV%), specificity (SP%) sensitivity (SN%) and Bonferroni corrected p-values are displayed. For regression models, variance explained (as R^2) and root mean squared error (RMSE) are displayed. Metabolites are abbreviated as: V4S: vanillin 4-sulfate, I3PA: indole 3-propionate, Sac: monosaccharide 1, PI: PI(16:0/18:1) and LPC: LPC(18:3). For the training models tested here, tree depth ranged from 25 to 101 nodes).

**Comparison**	Reduced candidates	Variables	Accuracy (%) (Lower - Upper)	AUC (%) (Lower - Upper)	SP (%)	SN (%)	NPV (%)	PPV (%)	adjusted p-value	R^2 (%)	RMSE
Within-participant	Final biomarker	5	94.7% (90.1 - 97.5)	99.2 (98.3 - 100)	96.3	93.1	92.9	96.4	<0.001	86.3	4.1
	No LPC	4	96.4% (92.4 - 98.7)	99.1 (98.2 - 100)	96.5	96.4	96.5	96.4	<0.001	84.6	4.4
	No PI	4	96.4% (92.4 - 98.7)	99.2 (98.4 - 100)	97.6	95.3	95.3	97.6	<0.001	88.1	3.8
	No Sac	4	92.3% (87.2 - 95.8)	98.3 (97 - 99.6)	91.9	92.8	92.9	91.7	<0.001	83.1	4.6
	No I3PA	4	97.6% (94.1 - 99.4)	99.8 (99.4 - 100)	97.6	97.6	97.6	97.6	<0.001	84.8	4.3
	No V4S	4	89.3% (83.7 - 93.6)	97.7 (96.2 - 99.3)	92.4	86.7	85.9	92.9	<0.001	80.2	4.9
	No LPC, No PI	3	96.4% (92.4 - 98.7)	99.3 (98.3 - 100)	96.5	96.4	96.5	96.4	<0.001	84.9	4.3
	No Sac, No LPC	3	92.9% (87.9 - 96.3)	98.4 (97.1 - 99.7)	92.0	93.9	94.1	91.7	<0.001	83.3	4.5
	No Sac, No PI	3	92.9% (87.9 - 96.3)	97.6 (95.7 - 99.4)	92.9	92.9	92.9	92.9	<0.001	80.0	5.0
	No Sac, No I3PA	3	95.3% (90.9 - 97.9)	98.7 (97.4 - 100)	93.3	97.5	97.6	92.9	<0.001	80.7	4.9
	No Sac, No V4S	3	87.6% (81.6 - 92.1)	95.5 (92.7 - 98.3)	86.4	88.9	89.4	85.7	<0.001	75.6	5.5
	No I3PA, No LPC	3	97.6% (94.1 - 99.4)	99.8 (99.6 - 100)	96.6	98.8	98.8	96.4	<0.001	86.4	4.1
	No I3PA, No PI	3	95.9% (91.7 - 98.3)	99.4 (98.8 - 100)	95.3	96.4	96.5	95.2	<0.001	82.0	4.7
	No V4S, No LPC	3	89.9% (84.4 - 94)	97.8 (96.2 - 99.4)	92.5	87.6	87.1	92.9	<0.001	81.8	4.7
	No V4S, No PI	3	91.1% (85.8 - 94.9)	97.2 (95.3 - 99.1)	93.8	88.8	88.2	94.0	<0.001	75.7	5.5
	No I3PA, No VS4	3	85.8% (79.6 - 90.7)	92.5 (88.6 - 96.4)	88.6	83.3	82.4	89.3	<0.001	68.2	6.3
	Only I3PA and V4S	2	89.9% (84.4 - 94)	96.6 (94.2 - 99)	92.5	87.6	87.1	92.9	<0.001	75.0	5.6
	Only V4S and PI	2	93.5% (88.7 - 96.7)	98.8 (97.8 - 99.9)	92.0	95.1	95.3	91.7	<0.001	80.6	4.9
	Only IPA and PI	2	88.8% (83 - 93.1)	94.8 (91.4 - 98.2)	88.4	89.2	89.4	88.1	<0.001	75.9	5.5
	Only Sac and PI	2	82.2% (75.6 - 87.7)	92.2 (88.4 - 96)	84.0	80.7	80.0	84.5	<0.001	68.8	6.2
	Only LPC and PI	2	78.7% (71.7 - 84.6)	87.9 (83 - 92.8)	81.0	76.7	75.3	82.1	<0.001	56.5	7.3
	Only Sac and V4S	2	96.4% (92.4 - 98.7)	99.4 (98.6 - 100)	95.4	97.6	97.6	95.2	<0.001	81.9	4.7
	Only LPC and VS4	2	92.3% (87.2 - 95.8)	97.3 (95.2 - 99.5)	89.1	96.1	96.5	88.1	<0.001	76.0	5.4
	Only Sac and I3PA	2	92.9% (87.9 - 96.3)	97.3 (95.4 - 99.3)	95.1	90.9	90.6	95.2	<0.001	74.9	5.6
	Only I3PA and LPC	2	83.4% (77 - 88.7)	92.8 (89 - 96.6)	82.0	85.0	85.9	81.0	<0.001	65.0	6.6
	Only Sac and LPC	2	83.4% (77 - 88.7)	90.3 (85.5 - 95)	87.0	80.4	78.8	88.1	<0.001	53.3	7.6
Between-participants	Final biomarker	5	79.3% (72.4 - 85.1)	89.1 (84.4 - 93.8)	76.0	83.6	85.9	72.6	<0.001	45.8	8.2
	No LPC	4	78.1% (71.1 - 84.1)	89.4 (84.8 - 94)	75.5	81.3	83.5	72.6	<0.001	47.0	8.1
	No PI	4	76.3% (69.2 - 82.5)	89.9 (85.5 - 94.4)	79.2	73.9	71.8	81.0	<0.001	47.3	8.1
	No Sac	4	75.7% (68.6 - 82)	84.3 (78.4 - 90.3)	75.6	75.9	76.5	75.0	<0.001	39.6	8.6
	No I3PA	4	72.8% (65.4 - 79.3)	84.7 (79.1 - 90.4)	68.9	78.8	83.5	61.9	0.044	38.1	8.7
	No V4S	4	79.9% (73 - 85.6)	86.6 (81 - 92.3)	75.2	86.8	89.4	70.2	<0.001	33.6	9.1
	No LPC, No PI	3	81.1% (74.3 - 86.7)	90.3 (85.8 - 94.7)	86.3	77.1	74.1	88.1	<0.001	46.4	8.1
	No Sac, No LPC	3	73.4% (66 - 79.9)	85.2 (79 - 91.5)	81.2	68.6	61.2	85.7	0.071	30.6	9.3
	No Sac, No PI	3	82.2% (75.6 - 87.7)	85.9 (80.1 - 91.8)	84.0	80.7	80.0	84.5	<0.001	35.5	8.9
	No Sac, No I3PA	3	66.9% (59.2 - 73.9)	73.5 (66 - 80.9)	69.3	64.9	61.2	72.6	0.098	19.7	10.0
	No Sac, No V4S	3	69.8% (62.3 - 76.6)	83.8 (77.8 - 89.9)	64.9	80.0	87.1	52.4	1	24.3	9.7
	No I3PA, No LPC	3	74% (66.7 - 80.4)	81.2 (74.8 - 87.6)	75.3	72.7	71.8	76.2	<0.001	30.0	9.3

Comparison	Reduced candidates	Variables	Accuracy (%) (Lower - Upper)	AUC (%) (Lower - Upper)	SP (%)	SN (%)	NPV (%)	PPV (%)	adjusted p-value	R^2 (%)	RMSE
	No I3PA, No PI	3	72.8% (65.4 - 79.3)	83.6 (77.7 - 89.5)	69.3	77.9	82.4	63.1	0.015	34.1	9.0
	No V4S, No LPC	3	79.3% (72.4 - 85.1)	88.1 (83.1 - 93)	78.4	80.2	81.2	77.4	<0.001	39.2	8.7
	No V4S, No PI	3	77.5% (70.5 - 83.6)	87.8 (82.5 - 93.2)	72.8	84.8	88.2	66.7	<0.001	26.8	9.5
	No I3PA, No VS4	3	75.1% (67.9 - 81.5)	83.8 (77.6 - 89.9)	69.0	87.5	91.8	58.3	0.638	22.3	9.8
	Only I3PA and V4S	2	74% (66.7 - 80.4)	84.1 (77.5 - 90.8)	84.7	68.2	58.8	89.3	0.445	19.9	10.0
	Only V4S and PI	2	62.7% (55 - 70)	71.5 (63.8 - 79.1)	66.2	60.4	52.9	72.6	1	-11.8	11.8
	Only IPA and PI	2	69.8% (62.3 - 76.6)	78.9 (72.2 - 85.7)	71.8	68.1	65.9	73.8	0.001	22.6	9.8
	Only Sac and PI	2	71.6% (64.2 - 78.3)	80.6 (74.1 - 87.2)	69.1	75.0	78.8	64.3	0.005	21.3	9.9
	Only LPC and PI	2	65.1% (57.4 - 72.2)	73.2 (65.7 - 80.7)	60.7	76.6	87.1	42.9	1	3.8	10.9
	Only Sac and V4S	2	71.6% (64.2 - 78.3)	78.4 (71.7 - 85.2)	77.6	67.6	61.2	82.1	0.079	27.0	9.5
	Only LPC and VS4	2	64.5% (56.8 - 71.7)	70.6 (62.8 - 78.5)	67.6	62.2	56.5	72.6	1	-0.4	11.1
	Only Sac and I3PA	2	77.5% (70.5 - 83.6)	86.3 (81 - 91.7)	75.3	80.3	82.4	72.6	<0.001	35.1	9.0
	Only I3PA and LPC	2	74% (66.7 - 80.4)	78.2 (71 - 85.4)	68.5	84.5	89.4	58.3	0.677	5.7	10.8
	Only Sac and LPC	2	70.4% (62.9 - 77.2)	79.9 (73.1 - 86.8)	65.8	79.3	85.9	54.8	1	9.3	10.6

Table S7.

Statistical results from individual t-tests comparing a 4 h block pre- and post- habitual sleep interval for clock time matched (2-6 h day 2 vs 2-6 h day 3) and evening/morning (12-16 h day 2 vs 2-6 h day 3) in the Matched Control experiment (5 participants n = 3 per treatment).

		Clock time Matched					Evening/Morning				
Metabolite	Participant	t-value	df	mean difference	p-value (fdr adjusted)	Trend	t-value	df	mean difference	p-value (fdr adjusted)	Trend
Vanillin 4-sulfate	1	2.7	2.9	-2.6	0.186	Not significant	1.7	2.1	-1.5	0.376	Not significant
	2	0.4	3.3	-0.3	0.809	Not significant	0.1	3.0	-0.1	0.969	Not significant
	3	-1.0	4.0	0.2	0.510	Not significant	-8.6	4.0	2.0	**0.021**	Increasing
	4	19.4	2.3	-1.7	**0.021**	Decreasing	-1.2	2.1	0.5	0.508	Not significant
	5	-8.9	3.7	2.4	**0.021**	Increasing	-6.2	4.0	1.8	**0.044**	Increasing
Indole 3-propionate	1	-2.5	3.8	0.7	0.183	Not significant	-3.1	3.4	0.8	0.163	Not significant
	2	5.0	3.8	-1.7	0.071	Not significant	3.7	3.9	-1.3	0.092	Not significant
	3	-5.1	3.0	1.3	0.071	Not significant	0.7	2.2	-0.4	0.661	Not significant
	4	-1.7	3.3	0.7	0.335	Not significant	-2.3	2.3	1.8	0.249	Not significant
	5	5.6	4.0	-1.4	0.051	Not significant	1.2	2.4	-0.2	0.505	Not significant
Monosaccharide 1	1	-1.0	2.3	0.6	0.510	Not significant	1.0	3.7	-0.9	0.510	Not significant
	2	-1.0	3.9	0.5	0.510	Not significant	1.8	2.1	-0.6	0.352	Not significant
	3	4.4	3.9	-0.7	0.071	Not significant	-0.6	2.6	0.2	0.703	Not significant
	4	-0.6	3.9	0.3	0.703	Not significant	0.5	4.0	-0.3	0.758	Not significant
	5	0.8	2.9	-0.5	0.615	Not significant	1.0	2.5	-0.6	0.510	Not significant
PI(16:0/18:1)	1	1.8	2.4	-0.9	0.335	Not significant	4.4	3.1	-1.3	0.092	Not significant
	2	3.1	3.4	-1.6	0.163	Not significant	2.7	3.1	-1.5	0.183	Not significant
	3	-3.2	2.8	1.1	0.183	Not significant	0.1	4.0	-0.1	0.969	Not significant
	4	-0.1	2.8	0.0	0.969	Not significant	1.9	3.9	-1.6	0.249	Not significant
	5	-1.1	2.3	0.4	0.510	Not significant	1.4	4.0	-0.8	0.376	Not significant
LPC(18:3)+CH2O3	1	2.6	3.2	-0.7	0.183	Not significant	2.4	2.2	-1.4	0.249	Not significant
	2	0.1	3.6	0.0	0.969	Not significant	4.8	3.4	-1.8	0.071	Not significant
	3	0.0	3.8	0.0	0.969	Not significant	2.1	3.4	-1.4	0.249	Not significant
	4	2.9	2.9	-1.7	0.183	Not significant	2.6	3.4	-0.8	0.183	Not significant
	5	2.1	3.0	-1.0	0.249	Not significant	4.4	3.9	-1.3	0.071	Not significant

REFERENCES AND NOTES

1. K. Uehli, A.J. Mehta, D. Miedinger, K. Hug, C. Schindler, E. Holsboer-Trachsler, J.D. Leuppi, N. Kunzli, Sleep problems and work injuries: A systematic review and meta-analysis. *Sleep Med. Rev.* **18**, 61–73 (2014).

2. S. Bioulac J.A. Micoulaud-Franchi, M. Arnaud, P. Sagaspe, N. Moore, F. Salvo, P. Philip, Risk of motor vehicle accidents related to sleepiness at the wheel: A systematic review and meta-analysis. *Sleep* **40**, zsx134 (2017).

3. J. L. Lim, D. F. Dinges, in *Molecular and Biophysical Mechanisms of Arousal, Alertness, and Attention*, D. W. Pfaff, B. L. Kieffer, Eds. (2008), vol. 1129, pp. 305–322.

4. C. Anderson, J. A. Horne, Sleepiness enhances distraction during a monotonous task. *Sleep* **29**, 573–576 (2006).

5. J. Collet, S. Ftouni, M. Clough, S.W. Cain, J. Fielding, C. Anderson, Differential impact of sleep deprivation and circadian timing on reflexive versus inhibitory control of attention. *Sci. Rep.* **10**, 7270 (2020).

6. R. Stickgold, M. P. Walker, Sleep-dependent memory consolidation and reconsolidation. *Sleep Med.* **8**, 331–343 (2007).

7. M. W. L. Chee, Limitations on visual information processing in the sleep-deprived brain and their underlying mechanisms. *Curr. Opin. Behav. Sci.* **1**, 56–63 (2015).

8. Y. Harrison, J. A. Horne, The impact of sleep deprivation on decision making: A review. *J. Exp. Psychol. Appl.* **6**, 236–249 (2000).

9. C. Anderson, D. L. Dickinson, Bargaining and trust: The effects of 36-h total sleep deprivation on socially interactive decisions. *J. Sleep Res.* **19**, 54–63 (2010).

10. J. Maccora, J. E. Manousakis, C. Anderson, Pupillary instability as an accurate, objective marker of alertness failure and performance impairment. *J. Sleep Res.* **28**, e12739 (2019).

11. C. Cajochen, S. B. Khalsa, J. K. Wyatt, C. A. Czeisler, D. J. Dijk, EEG and ocular correlates of circadian melatonin phase and human performance decrements during sleep loss. *Am. J. Physiol.* **277**, R640–R649 (1999).

12. J. M. Mullington, S.M. Abbott, J.E. Carroll, C.J. Davis, D.J. Dijk, D.F. Dinges, P.R. Gehrman, G.S. Ginsburg, D. Gozal, M. Haack, D.C. Lim, M. Macrea, A.I. Pack, D.T. Plante, J.A. Teske, P.C. Zee, Developing biomarker arrays predicting sleep and circadian-coupled risks to health. *Sleep* **39**, 727–736 (2016).

13. B. J. Wilhelm, Pupillography for the assessment of driver sleepiness. *Klin. Monbl. Augenheilkd.* **225**, 791–798 (2008).

14. C. Anderson, A. M. Chang, J. P. Sullivan, J. M. Ronda, C. A. Czeisler, Assessment of drowsiness based on ocular parameters detected by infrared reflectance oculography. *J. Clin. Sleep Med.* **09**, 907–920 (2013).

15. C. Cajochen, Alerting effects of light. *Sleep Med. Rev.* **11**, 453–464 (2007).

16. L. K. McCorry, Physiology of the autonomic nervous system. *Am. J. Pharm. Educ.* **71**, 78 (2007).

17. L. Seugnet, J. Boero, L. Gottschalk, S. P. Duntley, P. J. Shaw, Identification of a biomarker for sleep drive in flies and humans. *Proc. Natl. Acad. Sci. U.S.A.* **103**, 19913–19918 (2006).

18. M. Pajcin, S. Banks, J.M. White, J. Dorrian, G. M. Paech, C. Grant, K. Johnson, K. Tooley, J. Fidock, G.H. Kamimori, C.B. Della Vedova, Decreased salivary alpha-amylase levels are associated with performance deficits during sleep loss. *Psychoneuroendocrinology* **78**, 131–141 (2017).

19. C. S. Möller-Levet, S.N. Archer, G. Bucca, E.E. Laing, A. Slak, R. Kabiljo, J.C. Lo, N. Santhi, M. von Schantz, C.P. Smith, D.J. Dijk, Effects of insufficient sleep on circadian rhythmicity and expression amplitude of the human blood transcriptome. *Proc. Natl. Acad. Sci. U.S.A.* **110**, E1132–E1141 (2013).

20. E. S. Arnardottir, E.V. Nikonova, K.R. Shockley, A.A. Podtelezhnikov, R.C. Anafi, K.Q. Tanis, G. Maislin, D.J. Stone, J.J. Renger, C.J. Winrow, A.I. Pack, Blood-gene expression reveals reduced circadian rhythmicity in individuals resistant to sleep deprivation. *Sleep* **37**, 1589–1600 (2014).

21. R. Pellegrino, D.Y. Sunaga, C. Guindalini, R.C. Martins, D.R. Mazzotti, Z. Wei, Z.J. Daye, M.L. Andersen, S Tufik, Whole blood genome-wide gene expression profile in males after prolonged wakefulness and sleep recovery. *Physiol. Genomics* **44**, 1003–1012 (2012).

22. E. E. Laing, C. S. Möller-Levet, D. J. Dijk, S. N. Archer, Identifying and validating blood mRNA biomarkers for acute and chronic insufficient sleep in humans: A machine learning approach. *Sleep* **42**, zsy186 (2019).

23. D. S. Wishart, Emerging applications of metabolomics in drug discovery and precision medicine. *Nat. Rev. Drug Discov.* **15**, 473–484 (2016).

24. S. K. Davies, J.E. Ang, V.L. Revell, B. Holmes, A. Mann, F.P. Robertson, N. Cui, B. Middleton, K. Ackermann, M. Kayser, A.E. Thumser, F.I Raynaud, D.J. Skene, Effect of sleep deprivation on the human metabolome. *Proc. Natl. Acad. Sci. U.S.A.* **111**, 10761–10766 (2014).

25. E. C. P. Chua, G. Shui, I.T. Lee, P. Lau, L.C. Tan, S.C. Yeo, B.D. Lam, S. Bulchand, S.A. Summers, K. Puvanendran, S.G. Rozen, M.R. Wenk, J.J. Gooley, Extensive diversity in circadian regulation of plasma lipids and evidence for different circadian metabolic phenotypes in humans. *Proc. Natl. Acad. Sci. U.S.A.* **110**, 14468–14473 (2013).

26. L. K. Grant, S. Ftouni, B. Nijagal, D.P. De Souza, D. Tull, M.J. McConville, S.M.W. Rajaratnam, S.W. Lockley, C. Anderson, Circadian and wake-dependent changes in human plasma polar metabolites during prolonged wakefulness: A preliminary analysis. *Sci. Rep.* **9**, 4428 (2019).

27. C. M. Depner, D.T. Cogswell, P.J. Bisesi, R.R. Markwald, C. Cruickshank-Quinn, K. Quinn, E.L. Melanson, N. Reisdorph, K.P. Wright, Developing preliminary blood metabolomics-based biomarkers of insufficient sleep in humans. *Sleep* **43**, zsz321 (2020).

28. A. M. Weljie, P. Meerlo, N. Goel, A. Sengupta, M.S. Kayser, T. Abel, M.J. Birnbaum, D.F. Dinges, A. Sehgal, Oxalic acid and diacylglycerol 36:3 are cross-species markers of sleep debt. *Proc. Natl. Acad. Sci. U.S.A.* **112**, 2569–2574 (2015).

29. J. H. Marcus, M. R. Rosekind, Fatigue in transportation: NTSB investigations and safety recommendations. *Inj. Prev.* **23**, 232–238 (2017).

30. A. G. Verstraete, Oral fluid testing for driving under the influence of drugs: History, recent progress and remaining challenges. *Forensic Sci. Int.* **150**, 143–150 (2005).

31. S. Ganesan, M. Magee, J.E. Stone, M.D. Mulhall, A. Collins, M.E. Howard, S.W. Lockley, S.M.W. Rajaratnam, T.L. Sletten, The impact of shift work on sleep, alertness and performance in healthcare workers. *Sci. Rep.* **9**, 4635 (2019).

32. P. Knauth, Extended work periods. *Ind. Health* **45**, 125–136 (2007).

33. T. Roenneberg, K. V. Allebrandt, M. Merrow, C. Vetter, Social jetlag and obesity. *Curr. Biol.* **22**, 939–943 (2012).

34. P. T. George, The psycho-sensory wake drive—A power source for power naps and other common sleep-wake phenomena: A hypothesis. *Sleep Breath.* **22**, 41–48 (2018).

35. H. P. Van Dongen, M. D. Baynard, G. Maislin, D. F. Dinges, Systematic interindividual differences in neurobehavioral impairment from sleep loss: Evidence of trait-like differential vulnerability. *Sleep* **27**, 423–433 (2004).

36. M. J. Gasson, Y. Kitamura, W.R. McLauchlan, A. Narbad, A.J. Parr, E.L. Parsons, J. Payne, M.J. Rhodes, N.J. Walton, Metabolism of ferulic acid to vanillin. A bacterial gene of the enoyl-SCoA hydratase/isomerase superfamily encodes an enzyme for the hydration and cleavage of a hydroxycinnamic acid SCoA thioester. *J. Biol. Chem.* **273**, 4163–4170 (1998).

37. M. V. Selma, J. C. Espín, F. A. Tomás-Barberán, Interaction between phenolics and gut microbiota: Role in human health. *J. Agric. Food Chem.* **57**, 6485–6501 (2009).

38. T. Marjot, D. W. Ray, F. R. Williams, J. W. Tomlinson, M. J. Armstrong, Sleep and liver disease: A bidirectional relationship. *Lancet Gastroenterol. Hepatol.* **6**, 850–863 (2021).

39. Y. J. Chyan, B. Poeggeler, R.A. Omar, D.G. Chain, B. Frangione, J. Ghiso, M.A. Pappolla, Potent neuroprotective properties against the Alzheimer β-amyloid by an endogenous melatonin-related indole structure, indole-3-propionic acid. *J. Biol. Chem.* **274**, 21937–21942 (1999).

40. R. J. Reiter, J. M. Guerrero, J. J. Garcia, D. Acuna-Castroviejo, Reactive oxygen intermediates, molecular damage, and aging: Relation to melatonin. *Ann. N. Y. Acad. Sci.* **854**, 410–424 (1998).

41. D. G. Chain, Galasko, D.,. Neria, E., M. Pappolla, Bendheim, P.E., B. Poeggler, Antioxidants in the treatment of Alzheimer's disease: Breakthrough potential of indole-3propionic acid, in *Drug Discovery and Development for Alzheimer's Disease, 2000* (Springer Publishing Company, 2002), pp. 159–170.

42. P. E. Bendheim, Development of indole-3-propionic acid (OXIGON) for Alzheimer's disease. *J. Mol. Neurosci.* **19**, 213–217 (2002).

43. M. Tuomainen, J. Lindström, M. Lehtonen, S. Auriola, J. Pihlajamäki, M. Peltonen, J. Tuomilehto, M. Uusitupa, V.D. de Mello, K. Hanhineva, Associations of serum indolepropionic acid, a gut microbiota metabolite, with type 2 diabetes and low-grade inflammation in high-risk individuals. *Nutr. Diabetes* **8**, 35 (2018).

44. V. D. De Mello, J Paananen, J Lindström, M.A. Lankinen, L. Shi, J. Kuusisto, J Pihlajamäki, S. Auriola, M. Lehtonen, O Rolandsson, I.A. Bergdahl, E. Nordin, P. Ilanne-Parikka, S. Keinänen-Kiukaanniemi, R. Landberg, J.G. Eriksson, J. Tuomilehto, K. Hanhineva, M Uusitupa, Indolepropionic acid and novel lipid metabolites are associated with a lower risk of type 2 diabetes in the Finnish Diabetes Prevention Study. *Sci. Rep.* **7**, rep46337 (2017).

45. C. Chimerel, E. Emery, D.K. Summers, U. Keyser, F.M. Gribble, F. Reimann, Bacterial metabolite indole modulates incretin secretion from intestinal enteroendocrine L cells. *Cell Rep.* **9**, 1202–1208 (2014).

46. L. Shi, S.J. Chen, M.Y. Ma, Y.P. Bao, Y. Han, Y.M. Wang, J. Shi, M.V. Vitiello, L. Lu, Sleep disturbances increase the risk of dementia: A systematic review and meta-analysis. *Sleep Med. Rev.* **40**, 4–16 (2018).

47. J. Van Erum, D. Van Dam, P. P. De Deyn, Sleep and Alzheimer's disease: A pivotal role for the suprachiasmatic nucleus. *Sleep Med. Rev.* **40**, 17–27 (2018).

48. O. Itani, M. Jike, N. Watanabe, Y. Kaneita, Short sleep duration and health outcomes: A systematic review, meta-analysis, and meta-regression. *Sleep Med.* **32**, 246–256 (2017).

49. E. C. P. Chua, G. H. Shui, A. Cazenave-Gassiot, M. R. Wenk, J. J. Gooley, Changes in plasma lipids during exposure to total sleep deprivation. *Sleep* **38**, 1683–1691 (2015).

50. R. Dallmann, A. U. Viola, L. Tarokh, C. Cajochen, S. A. Brown, The human circadian metabolome. *Proc. Natl. Acad. Sci. U.S.A.* **109**, 2625–2629 (2012).

51. E. Maury, K. M. Ramsey, J. Bass, in *Metabolic Basis of Obesity* (2011), pp. 229–255.

52. G. Di Paolo, P. De Camilli, Phosphoinositides in cell regulation and membrane dynamics. *Nature* **443**, 651–657 (2006).

53. S.-H. Law, M.L. Chan, G.K. Marathe, F. Parveen, C.H. Chen, L.Y. Ke, An updated review of lysophosphatidylcholine metabolism in human diseases. *Int. J. Mol. Sci.* **20**, 1149 (2019).

54. V.-P. Mäkinen, M. Ala-Korpela, Metabolomics of aging requires large-scale longitudinal studies with replication. *Proc. Natl. Acad. Sci. U.S.A.* **113**, E3470 (2016).

55. O. Robinson, C. E. Lau, Measuring biological age using metabolomics. *Aging* **12**, 22352–22353 (2020).

56. P. Vidafar, J.J. Gooley, A.C. Burns, S.M.W. Rajaratnam, M. Rueger, E. Van Reen, C.A. Czeisler, S.W. Lockley, S.W. Cain, Increased vulnerability to attentional failure during acute sleep deprivation in women depends on menstrual phase. *Sleep* **41**, zsy098 (2018).

57. J. M. Mullington, Please forgive our appearance while under biomarker construction. *Sleep* **42**, zsy267 (2019).

58. W. R. McMahon, S. Ftouni, S.P.A. Drummond, P. Maruff, S.W. Lockley, S.M.W. Rajaratnam, C. Anderson, The wake maintenance zone shows task dependent changes in cognitive function following one night without sleep. *Sleep*, **41**, zsy148 (2018).

59. *Australian Dietary Guidelines* (2013).

60. R. J. Stewart, L. Whitehead, B. Nijagal, B.E. Sleebs, G. Lessene, M.J. McConville, K.L. Rogers, C.J. Tonkin, Analysis of Ca^{2+} mediated signaling regulating toxoplasma infectivity reveals complex relationships between key molecules. *Cell. Microbiol.* **19**, cmi.12685 (2017).

61. R. Tautenhahn, C. Bottcher, S. Neumann, Highly sensitive feature detection for high resolution LC/MS. *BMC Bioinformatics* **9**, 504 (2008).

62. T. L. Sletten, S. Vincenzi, J. R. Redman, S. W. Lockley, S. M. Rajaratnam, Timing of sleep and its relationship with the endogenous melatonin rhythm. *Front. Neurol.* **1**, 137 (2010).

63. D. J. Creek, A. Jankevics, K. E. V. Burgess, R. Breitling, M. P. Barrett, IDEOM: An Excel interface for analysis of LC-MS-based metabolomics data. *Bioinformatics*. **28**, 1048–1049 (2012).

64. L. W. Sumner, A. Amberg, D. Barrett, M. H. Beale, R. Beger, C. A. Daykin, Teresa W-M Fan, O. Fiehn, R. Goodacre, J. L. Griffin, T. Hankemeier, N. Hardy, J. Harnly, R. Higashi, J. Kopka, A. N. Lane, J. C. Lindon, P. Marriott, A. W. Nicholls, M. D. Reily, J. J. Thaden, M. R. Viant, Proposed minimum reporting standards for chemical analysis Chemical Analysis Working Group (CAWG) Metabolomics Standards Initiative (MSI). *Metabolomics* **3**, 211–221 (2007).

65. T. M. Deist, F.J.W.M. Dankers, G. Valdes, R. Wijsman, I.C. Hsu, C. Oberije, T. Lustberg, J. van Soest, F. Hoebers, A. Jochems, I. El Naqa, L. Wee, O. Morin, D.R. Raleigh, W. Bots, J.H. Kaanders, J. Belderbos, M. Kwint, T. Solberg, R. Monshouwer, J. Bussink, A. Dekker, P. Lambin, Machine learning algorithms for outcome prediction in (chemo)radiotherapy: An empirical comparison of classifiers. *Med. Phys.* **45**, 3449–3459 (2018).

66. S. I. Dimitriadis, D. Liparas, Alzheimer's Disease Neuroimaging Initiative; How random is the random forest? Random forest algorithm on the service of structural imaging biomarkers for Alzheimer's disease: From Alzheimer's disease neuroimaging initiative (ADNI) database. *Neural Regen. Res.* **13**, 962–970 (2018).

67. Y. Fan, T.B. Murphy, J.C. Byrne, L. Brennan, J.M. Fitzpatrick, R.W. Watson, Applying random forests to identify biomarker panels in serum 2D-DIGE data for the detection and staging of prostate cancer. *J. Proteome Res.* **10**, 1361–1373 (2011).

68. Z. Yan, J. Li, Y. Xiong, W. Xu, G. Zheng, Identification of candidate colon cancer biomarkers by applying a random forest approach on microarray data. *Oncol. Rep.* **28**, 1036–1042 (2012).

69. A. Liaw, M. Wiener, Classification and regression by randomForest. *R News* **2**, 18–22 (2002).

70. R. Genuer, J. M. Poggi, C. Tuleau-Malot, VSURF: An R package for variable selection using random forests. *R J.* **7**, 19–33 (2015).

71. D. Bates, M. Mächler, B. Bolker, S. Walker, Fitting linear mixed-effects models using lme4. *J. Stat. Softw.* **67**, 1–48 (2015).
